# S2k-Leitlinie zur Diagnostik und Therapie des Zoster und der Postzosterneuralgie

**DOI:** 10.3205/id000045

**Published:** 2020-03-12

**Authors:** Gerd E. Gross, Lisa Eisert, Hans Wilhelm Doerr, Helmut Fickenscher, Markus Knuf, Philip Maier, Matthias Maschke, Rainer Müller, Uwe Pleyer, Michael Schäfer, Cord Sunderkötter, Ricardo N. Werner, Peter Wutzler, Alexander Nast

**Affiliations:** 1 Universitätsmedizin Rostock, Universitätsklinik für Dermatologie und Venerologie, Rostock, Deutschland; 2 Vivantes Klinikum Neukölln, Klinik für Dermatologie und Venerologie, Berlin, Deutschland; 3 Universitätsklinikum Frankfurt, Institut für Medizinische Virologie, Frankfurt (Main), Deutschland; 4 Christian-Albrechts-Universität zu Kiel und Universitätsklinikum Schleswig-Holstein, Institut für Infektionsmedizin, Kiel, Deutschland; 5 Helios Dr. Horst Schmidt Kliniken Wiesbaden, Kinder- und Jugendklinik, Wiesbaden, Deutschland; 6 Universitätsklinikum Freiburg, Klinik für Augenheilkunde, Freiburg, Deutschland; 7 Krankenhaus der Barmherzigen Brüder Trier, Klinik für Neurologie, Neurophysiologie und neurologische Frührehabilitation, Trier, Deutschland; 8 Medizinische Fakultät Carl Gustav Carus der Technischen Universität Dresden, Klinik und Poliklinik für Hals-, Nasen- und Ohrenheilkunde, Dresden, Deutschland; 9 Charité – Universitätsmedizin Berlin, corporate member of Freie Universität Berlin, Humboldt-Universität zu Berlin, and Berlin Institute of Health, Augenklinik, Berlin, Deutschland; 10 Charité – Universitätsmedizin Berlin, corporate member of Freie Universität Berlin, Humboldt-Universität zu Berlin, and Berlin Institute of Health, Klinik für Anästhesiologie und operative Intensivmedizin, Berlin, Deutschland; 11 Universitätsklinikum Halle (Saale), Universitätsklinik und Poliklinik für Dermatologie und Venerologie, Halle (Saale), Deutschland; 12 Charité – Universitätsmedizin Berlin, corporate member of Freie Universität Berlin, Humboldt-Universität zu Berlin, and Berlin Institute of Health, Department of Dermatology, Venereology and Allergy, Division of Evidence-based Medicine (dEBM), Berlin, Deutschland; 13 Universitätsklinikum Friedrich-Schiller-Universität Jena, Virologie, Jena, Deutschland

**Keywords:** Herpes Zoster, Gürtelrose, Herpes Zoster ophthalmicus, Herpes Zoster oticus, Neuralgie, Zoster-assoziierte Schmerzen, Varicella-Zoster-Virus, Varizellen, Aciclovir, herpes zoster, shingles, herpes zoster opthalmicus, herpes zoster oticus, neuralgia, zoster associated pain, varicella zoster virus, varicella, acyclovir

## Abstract

Diese Leitlinie richtet sich an Dermatologen, Ophthalmologen, HNO-Ärzte, Pädiater, Neurologen, Virologen sowie Infektiologen, Anästhesisten und Allgemeinmediziner in Klinik und Praxis und dient zur Information für andere medizinische Fachrichtungen, die an der Behandlung des Zoster beteiligt sind. Darüber hinaus soll die Leitlinie Kostenträgern und politischen Entscheidungsträgern zur Orientierung dienen. Die Leitlinie wurde im formellen Konsensusverfahren (S2k) von Dermatologen, Virologen/Infektiologen, Ophthalmologen, HNO-Ärzten, Neurologen, Pädiatern und Anästhesisten/Schmerzmedizinern erstellt.

Die Leitlinie stellt einen Überblick über die klinische und molekulare Diagnostik sowie den Antigennachweis, die Antikörperkultur und Viruskultur dar. Diagnostisch besondere Situationen und komplizierte Verläufe der Erkrankung finden ebenfalls Berücksichtigung. Die antivirale Therapie des Zoster und der Postzosterneuralgie wird im Allgemeinen und für besondere Situationen dargelegt. Detaillierte Angaben zur Schmerzbehandlung finden Erwähnung und sind in einer Übersicht dargestellt. Ebenso werden die lokaltherapeutischen Maßnahmen thematisiert.

## Abstract (en)

This guideline is aimed at registrars and consultants in dermatology, ophthalmology, ENT, pediatrics, neurology, virology as well as infectiology, anaesthesia and generell medicine as well as policymakers and payers and purchasers of care. It was developed by dermatologists, virologists, ophthalmologists, ENT physicians, neurologists, pediatrician and anesthetists using a formal consensus process (S2k).

The guideline provides an overview of clinical and molecular diagnostics as well as antigen detection, antibody culture and viral culture. Diagnostic special situations and complicated courses of the disease are also considered. The antiviral therapy of zoster and postzoster neuralgia is presented in general and for special situations. Detailed information on the treatment of pain is mentioned and presented in an overview. Likewise, the local therapeutic measures are discussed.

## 0 Vorbemerkung

Um die Disseminierung der Leitlinie in den verschiedenen medizinischen Fächern zu fördern, wird dieser Artikel ebenfalls im Journal der Deutschen Dermatologischen Gesellschaft publiziert. Dort ist der Artikel veröffentlicht unter Creative Commons Lizenz CC BY-NC. Die Zählung der Tabellen und References weicht zum Teil ab.

## 1 Klinische Einleitung

Der Zoster (Gürtelrose) wird durch die Reaktivierung des latent in sensorischen Spinal- und Hirnnervenganglien persistierenden Varicella-Zoster-Virus (VZV) verursacht. Der international gültige Fachbegriff lautet Herpes zoster (so im Englischen, auch im Französischen „herpès zoster“; zoster altgriechisch für Gürtel). Im Deutschen hat sich auch der verkürzte Fachbegriff „Zoster“ einbgebürgert, der auch in der medizinischen Terminologie wie „postzosterisch“ Eingang gefunden hat. Der Einfachheit halber wird in dieser Leitlinie nur der Begriff Zoster gebraucht. 

Die Erstinfektion mit VZV erfolgt überwiegend im Kindesalter und äußert sich in den meisten Fällen durch ein generalisiertes Exanthem (sog. Windpocken, Varizellen). Der Zoster ist eine neurokutane Viruskrankheit, die in jedem Lebensalter auftreten kann, signifikant zunehmend jedoch erst ab dem 50. Lebensjahr. Dabei handelt sich um eines der häufigsten, akuten Krankheitsbilder der Haut. Mit dem Problem schwieriger Zosterfälle werden Ärzt(inn)e(n) vieler Fachrichtungen konfrontiert. Dies ist national und international auf interdisziplinären Konsensuskonferenzen diskutiert worden [1], [2].

Die Latenz von VZV wird durch die körpereigene VZV-spezifische Immunabwehr gewährleistet. Wenn die Kontrolle durch Alterungsprozesse (Immunseneszenz) oder Defizienz der zellulären Immunität bei malignen Lymphomen, bei HIV-Infektionen oder unter immunsuppressiver Therapie nachlässt, können die latent persistierenden VZV erneut aktiv replizieren. Ein Sonderfall ist der Zoster als Folge einer pränatalen VZV-Infektion beim immunologisch noch nicht voll kompetenten Neugeborenen. In der Folge kommt es zu Entzündungen und Nekrosen in einem oder in mehreren betroffenen Ganglien [3]. Drei bis fünf Tage nach Beginn der VZV-Reaktivierung entwickelt sich in der Regel der typische, dermatomale halbseitige Hautausschlag, der als Gürtelrose bekannt ist.

Hinweisend ist ein halbseitiges, umschriebenes Exanthem, in dem sich gruppiert stehende Bläschen entwickeln. In 80% der Zosterfälle geht der Hautmanifestation ein Prodromalstadium voraus. Die uneinheitliche Symptomatik in dieser Phase mit Allgemeinbeschwerden und meist leichten bis mäßigen Schmerzen im befallenen Dermatom kann lokalisationsabhängig zu Fehldiagnosen wie z.B. Cholezystitis, Herzinfarkt, Glaukom und anderen führen. Besonders gilt dies für den sogenannten Herpes Zoster sine herpete. Hier entwickeln sich nach der Prodromalsymptomatik dermatomale Schmerzen, allerdings ohne Zostereffloreszenzen. Das charakteristische Zosterexanthem betrifft in der Regel ein einziges Dermatom. Oft werden aber mehrere Segmente überlappend befallen. Selten kommt es zu multisegmentalem Zoster auf beiden Körperseiten. Der Zoster ist überwiegend thorakal lokalisiert. Mit zunehmendem Alter wird der Zoster im Kopfbereich häufiger diagnostiziert. Bei ausgeprägter Beschwerdesymptomatik ist eine Behandlung unter stationären Bedingungen erforderlich. Trotz Abheilung des Ausschlags können starke Zosterschmerzen als Folge einer Ganglionitis persistieren. Schmerzen, die länger als drei Monate andauern, werden definitionsgemäß als postzosterische Neuralgie (PZN) bezeichnet. Für die akute Behandlung ist wichtig, ob es sich um einen nozizeptiven, neuropathischen oder gemischt nozizeptiv-neuropathischen Schmerz handelt (siehe Kapitel 5.2.1), unabhängig davon, wann er begonnen hat. Die mit Zoster einhergehenden, anhaltenden Schmerzen sind die häufigste Komplikation des Zoster. Wie der Zoster weist auch die PZN eine Altersabhängigkeit auf. Die globalen Inzidenzraten belaufen sich auf 3–5 pro 1.000 Personenjahre (PJ) [4]. Die alterspezifische Inzidenz weist einen steilen Anstieg nach dem 50. Lebensjahr auf, mit Werten von 5/1.000 PJ für die 50–60-Jährigen, 6–7/1.000 PJ für die 70–80-Jährigen und bis zu 10/1.000 PJ für die über 90-Jährigen [4]. Bei etwa 20% der über 60-jährigen Patienten persistieren die Komplikationen länger als ein Jahr [5]. Zu den Komplikationen des Zoster am Nervensystem zählen auch passagere segmentale Lähmungen wie Bauchwandhernien, Harnblasendysfunktionen sowie Enzephalitis und Meningitis [6].

Komplikationen des Zoster am Auge umfassen Entzündungen, Keratitis, Uveitis, Glaukom sowie die akute und chronische Retinanekrose. Hier kann eine Vaskulitis oder eine Meningitis vorausgehen [7]. Komplikationen an der Haut reichen von bakteriellen Sekundärinfektionen, langer Persistenz der Hautveränderungen, bis zu Dissemination mit varizellenartigem Haut- und Organbefall bei immundefizienten Patienten [8]. Vor allem in den ersten vier Wochen nach dem Zosterexanthem besteht auch ein Risiko für Vaskulopathien und Schlaganfall [9], [10]. 

Der Zoster tritt sporadisch auf. Die Lebenszeitprävalenz beträgt weltweit 25–50% [11]. Die Häufigkeit steigt mit zunehmendem Alter, ab dem 50. Lebensjahr und auch bei geschwächtem Immunsystem, sei es erkrankungsbedingt oder als Therapiefolge [12]. Zwischen dem 10. und 49. Lebensjahr liegt die Häufigkeit des Zoster bei vier Erkrankungen pro 1000 PJ. Ab dem 50. Lebensjahr steigt die Erkrankungshäufigkeit mit jedem Jahr kontinuierlich an bis auf ca. 14 Erkrankungen pro 1.000 PJ im Alter von 75 Jahren. Danach bleibt die Inzidenz stabil [13]. Laut Beobachtungen von Hope-Simpson 1965 [14] erkranken Menschen, die das 85. Lebensjahr erreichen, mindestens einmal an Zoster. Mit dem demographischen Wandel und steigenden Zahlen immunsupprimierter Menschen in Deutschland ist in Zukunft mit einem weiteren Anstieg des Zoster zu rechnen. Aktuell liegt die Anzahl der Erkrankungsfälle in unserem Land geschätzt bei ca. 400.000/Jahr. Für die Europäische Union gehen Schätzungen von ca. zwei Millionen Erkrankungsfällen pro Jahr aus. Davon müssen mindestens 10% wegen einer Komplikation stationär in einem Krankenhaus aufgenommen werden. 

Die verfügbaren Therapiestrategien haben das Ziel, die Schmerzen in der Akutphase des Zoster zu lindern, die Ausdehnung und Dauer des Zosterexanthems zu begrenzen und die Schmerzen (u.a. PZN) sowie andere akute und chronische Komplikationen zu verhindern bzw. abzuschwächen. Die Therapie des akuten Zoster besteht aus der so früh wie möglich einsetzenden systemischen antiviralen Chemotherapie, kombiniert mit einer lokalen antiseptischen Therapie und konsequenter Schmerztherapie [6]. Die systemische antivirale Therapie erfolgt entweder mit dem Nukleosidanalogon Aciclovir (oral oder parental) oder mit einem der anderen oral verabreichten Nukleosidanaloga Valaciclovir, Famciclovir oder Brivudin.

Die frühestmögliche Behandlung der akuten Schmerzsymptomatik wird angestrebt, damit eine mögliche Chronifizierung der Schmerzen verhindert wird. Sie erfolgt entsprechend der Schmerzintensität nach dem WHO-Stufenschema mit nichtsteroidalen Antiphlogistika, z.T. mit Opioiden. Koanalgetika wie Antidepressiva und Antikonvulsiva sind ergänzend hilfreich. Bei fehlender oder nicht konsequenter multimodaler Therapie ist in vielen Fällen eine PZN möglich (siehe Kapitel 5.2 und 5.3).

Wegen der zunehmenden Zahl der Menschen in Deutschland mit alters-, krankheits- oder therapiebedingten Einschränkungen des Immunsystems steigt das Populationsrisiko für Zoster und PZN stark an. In vielen Fällen, vor allem bei älteren Menschen und bei Abwehrgeschwächten führt der Zoster zu einer Herabsetzung der Lebensqualität [15]. 

Aufgrund der gesamten Problematik des Zoster und seiner Komplikationen ist die Prävention mithilfe von Impfstoffen dringend indiziert. Auch Daten über die mögliche Rolle von VZV bei Vaskulopathie, Schlaganfall [9], [10] und bei Riesenzellarteriitis [16] unterstützen die Fortführung intensiver Entwicklung und breiter Anwendung von Zosterimpfstoffen. Primär verfolgt die Zosterimpfung das Ziel, eine Reaktivierung von VZV und damit den Zoster, PZN und andere Komplikationen zu unterdrücken. Der in Deutschland zugelassene attenuierte Lebendimpfstoff Zostavax^®^ wird von der STIKO aufgrund der eingeschränkten Wirksamkeit und seiner begrenzten Wirkdauer nicht als Standardimpfung empfohlen [17], [18].

Neben dem bereits vor einigen Jahren zugelassenen Lebendimpfstoff Zostavax^®^ ist ein neuer, rekombinanter Zosterimpfstoff, der sogenannte adjuvantierte Subunit-Totimpfstoff, Shingrix^®^, entwickelt worden. Dieser Impfstoff enthält als VZV-spezifisches Antigen das rekombinante VZV-Glykoprotein E (VZV-gE), zusammen mit dem Adjuvans, d.h. Immunverstärker, ASO1_β_. Shringrix^®^ wird intramuskulär in zwei Dosen im Abstand von zwei Monaten (maximal sechs Monaten) gegeben. Der Impfstoff steigert sowohl die zelluläre als auch die humorale Abwehr [19], [20].

Seit März 2018 ist dieser rekombinante, adjuvantierte Subunit Totimpfstoff zur Verhinderung des Zoster und der PZN für Personen ab dem 50. Lebensjahr in Deutschland zugelassen. In zwei Zulassungsstudien zeigte dieser Impfstoff bei 15.411 Personen im Alter ≥50 Jahren und bei über 13.900 Personen ≥70 Jahren eine Wirksamkeit von ≥90% auf Zoster und von ≥89% auf chronische Schmerzen und PZN. Die Wirksamkeit hielt bisher über vier Jahre an [20]. Die zelluläre und die humorale Immunantwort konnte unabhängig vom Alter der Probanden über neun Jahre gleichbleibend nachgewiesen werden [21]. Shingrix^®^ ist seit Mai 2018 in Deutschland verfügbar. Die STIKO empfiehlt die Impfung mit dem adjuvantierten Herpes-zoster-Subunit-Totimpfstoff zur Verhinderung von Zoster und der PZN allen Personen ab einem Alter von 60 Jahren (Standardimpfung).

Desweiteren empfiehlt die STIKO die Impfung mit dem Herpes-zoster-Subunit-Totimpfstoff gegen Zoster und PZN allen Personen ab einem Alter von 50 Jahren, die wegen einer erhöhten gesundheitlichen Gefährdung infolge einer Grundkrankheit oder wegen einer Immunsuppression ein erhöhtes Risiko für den Zoster und für PZN haben (Indikationsimpfung). Wirksamkeit und Sicherheit wurden in mehreren Studien für Patienten mit eingeschränktem Immunsystem nachgewiesen.

Für Patienten mit einer Grundkrankheit, wie z.B. rheumatoider Arthritis, chronischer Nierenerkrankung, chronisch obstruktiver Lungenerkrankung oder Diabetes mellitus, die in den Impfstoff-Zulassungsstudien eingeschlossen waren, zeigten stratifizierte Datenanalysen zur Wirksamkeit des Impfstoffs in diesen Gruppen keinen Unterschied im Vergleich zur Gesamtwirksamkeit.

Die Kostenübernahme für den Subunit Totimpfstoff Shingrix^®^ bei über 60-Jährigen (und bei über 50-Jährigen mit Immundefizienz) durch die gesetzlichen Krankenkassen wurde am 07.03.2019 vom Gemeinsamen Bundesauschuss (GBA) beschlossen.

## 2 Hinweise zur Anwendung von Leitlinien

Leitlinien stellen systematisch entwickelte Hilfen für klinisch relevante Beratungs- und Entscheidungssituationen dar. Während der Entwicklung einer Leitlinie kann nur eine beschränkte Auswahl standardisierter klinischer Situationen berücksichtigt werden. Empfehlungen klinischer Leitlinien haben keinen rechtlich verbindlichen Charakter; in spezifischen Situationen kann und muss unter Umständen von den hierin enthaltenen Empfehlungen abgewichen werden [22]. Die Umsetzung von Empfehlungen einer Leitlinie in spezifischen klinischen Situationen muss stets unter Berücksichtigung sämtlicher individueller patientenrelevanter Gegebenheiten (z.B. Komorbidität, Komedikation, Kontraindikationen) geprüft werden [23].

Die Medizin ist als Wissenschaft ständigen Entwicklungen unterworfen. Nutzer der Leitlinie werden aufgefordert, sich über neue Erkenntnisse nach Veröffentlichung der Leitlinie zu informieren. Anwender dieser Leitlinie sind zudem angehalten, durch sorgfältige Prüfung der Angaben sowie unter Berücksichtigung der Produktinformationen der Hersteller zu überprüfen, ob die gegebenen Empfehlungen bezüglich der Art der Durchführung der Interventionen, zu berücksichtigender Kontraindikationen, Arzneimittelinteraktionen etc. sowie hinsichtlich der Zulassungs- und Erstattungssituation vollständig und aktuell sind. 

Die in der Arbeit verwandten Personen- und Berufsbezeichnungen sind gleichwertig für beide Geschlechter gemeint, auch wenn sie nur in einer Form genannt werden.

## 3 Methodik

Diese Leitlinie wurde auf Grundlage der europäischen Leitlinen „European consensus-based (S2k) Guideline on the Management of Herpes Zoster – guided by the European Dermatology Forum (EDF) in cooperation with the European Academy of Dermatology and Venereology (EADV), Part 1: Diagnosis“ [1] und „European consensus-based (S2k) Guideline on the Management of Herpes Zoster – guided by the European Dermatology Forum (EDF) in cooperation with the European Academy of Dermatology and Venereology (EADV), Part 2: Treatment“ [2] erstellt. Die Genehmigung zur Adaptierung und partiellen Übernahme liegt vom Erstautor der Quellleitlinien Dr. med. Ricardo Werner vor.

Die Erstellung der Leitlinie erfolgte im Auftrag des Deutschen Herpes Management Forums (DHMF) der Paul-Ehrlich-Gesellschaft für Chemotherapie e.V.

Ein individuelles Abwägen und Gewichten einzelner für die Therapieauswahl relevanter Aspekte muss immer vorgenommen werden. Die Entscheidung für oder gegen eine Therapie bleibt eine Einzelfallentscheidung. Diese Leitlinie bietet wissenschaftlich begründete Entscheidungshilfen zur Auswahl einer geeigneten Therapie und ist eine medizinische Hilfe zum optimalen Einsatz des gewählten Therapieverfahrens.

### 3.1 Generierung von Empfehlungen; Empfehlungsstärken, Wording und Symbolik

Die Erstellung der Leitlinie erfolgte entsprechend der methodischen Vorgaben der Arbeitsgemeinschaft der Wissenschaftlichen Medizinischen Fachgesellschaften e.V. (AWMF).

**Abbildung 1:**
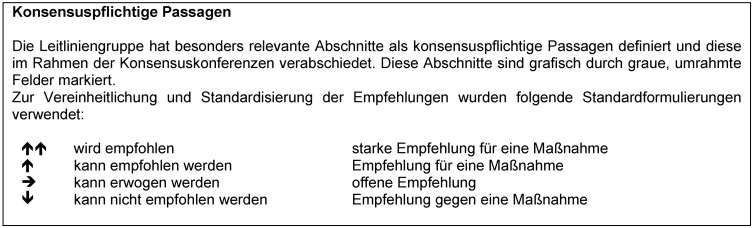
Abbildung 1: Empfehlungsstärken – Wortwahl, Symbolik und Interpretation

Text- und Empfehlungsentwürfe der Leitlinienkapitel wurden durch die Autoren ausgearbeitet und anschließend der Leitliniengruppe per Email vorgelegt. Bei der Ableitung der Empfehlungen wurden die in Abbildung 1 (Fig. 1) dargestellten Empfehlungsgrade unterschieden, welche die Stärke der Empfehlungen ausdrücken. Zur Standardisierung der Empfehlungen der Leitlinie wurden einheitliche Formulierungen verwendet. Eine Darstellung der Wortwahl, Symbolik und Hinweise zur Interpretation der Empfehlungsstärken ist in Abbildung 1 (Fig. 1) dargestellt.

## 4 Diagnostik

### 4.1 Klinische Diagnose

Der klassische klinische Befund eines Zoster stellt sich als unilaterale, auf ein Dermatom [14], [24], [25] begrenzte Hautveränderungen dar, die sich in der Regel von erythematösen Maculae und Papeln zu Vesiculae und Pusteln und jeweils nach fünf bis sieben Tagen zu Krusten entwickeln. Typischerweise nehmen die Effloreszenzen innerhalb von 24 bis 72 Stunden zu und breiten sich strahlenförmig über das Dermatom aus. Nicht immer ist das gesamte Dermatom betroffen, je nach Lokalisation können jedoch auch anhängende Dermatome beteiligt sein. Für gewöhnlich werden die Läsionen durch Schmerzen und Missempfindungen wie Juckreiz, Parästhesien, Dysästhesien oder Taubheitsgefühle begleitet, die meist schon einige Tage vor den Hauterscheinungen beginnen. Die Schmerzsymptomatik in der Prodromalphase führt häufig zu einem breiten Spektrum von Fehldiagnosen, die lokalisationsabhängig als Herzinfarkt, Cholecystitis, Zahnschmerzen etc. fehlinterpretiert werden [6], [26], [27]. Die Schmerzqualität wird häufig als brennend, stechend und pulsierend charakterisiert (siehe Kapitel 5.2, [28], [29], [30]).

Eine lokale Lymphadenopathie kann auftreten. Bei Patienten, die Antikoagulanzien oder Antiaggreganzien einnehmen oder sich unter einer Langzeittherapie mit Kortikosteroiden befinden, kann es zu hämorrhagischen Läsionen kommen. Der Zoster betrifft am häufigsten thorakale Dermatome (55%), gefolgt von der Trigeminusregion (20%), zervikalen (11%), lumbalen (13%) und sakralen (2%) Dermatomen [24]. In manchen Fällen treten benachbarte oder nicht benachbarte multisegmentale Befälle auf, sehr selten kann es auch zu einem bilateralen Zoster kommen [31]. 

Die rein klinische Diagnose des Zoster hat in Untersuchungen, in denen die Diagnose im Labor bestätigt wurde, eine Spezifität von 60–90%, je nach Ausprägung und Lokalisation. Differentialdiagnostisch muss an Herpes-simplex-Virus-Infektionen (HSV1 vor allem im Kopf-/Halsbereich, HSV2 insbesondere im Lumbosakralbereich) sowie zosteriforme dermatologische Erkrankungen gedacht werden [6]. 

Aufgrund der zosterspezifischen antiviralen Therapie und der heute frühzeitig eingesetzten begleitenden Schmerztherapie ist eine möglichst zeitnahe labordiagnostische Bestätigung anzustreben (siehe unten). Dies gilt insbesondere, wenn

- die typische Prodromalphase in der Anamnese fehlt.
- die Läsionen über mehr als ein Dermatom oder Mittellinien überschreitend lokalisiert sind.
- ein anderer als der thorakale Bereich betroffen ist.
- der zeitliche Verlauf untypisch ist.
- die betroffene Person schon einmal einen Zoster hatte.
- die betroffene Person gegen Varizellen geimpft wurde.

### 4.2 Molekulare Diagnostik

Der molekulare Nachweis von VZV-DNA aus Abstrichen gilt heute als Goldstandard für die Labordiagnostik der VZV-Infektion. Die modernen Realtime-PCR-Verfahren haben bei korrekter Durchführung und kontaminationsfreien Bedingungen eine Sensitivität und Spezifität nahe 100%. Neue isothermale und Helikase-Amplifikationsmethoden sind in der Sensitivität und Spezifität der klassischen PCR noch leicht unterlegen [32], [33], [34], [35], [36]. Für die Abstriche werden geflockte Tupfer oder Nylontupfer mit stabilisierendem Transportmedium empfohlen, die häufig von den Laboren für die Diagnostik zur Verfügung gestellt werden. Zum Nachweis der VZV-DNA im Abstrich muss kein flüssigkeitsgefülltes Bläschen vorhanden sein. Auch im makulopapulösen Anfangsstadium sowie in Abheilung befindlichen Läsionen kann die Virus-DNA in aller Regel zuverlässig detektiert werden. Bei Krusten sollte der oberflächliche Schorf entfernt werden und am Krustengrund abgestrichen werden. Im mit den Tupfern gelieferten Flüssigmedium sind die Abstriche über mehrere Tage stabil und können auf dem normalen Postweg ins Labor transportiert werden. 

Bei Verdacht auf ZNS-Beteiligung bzw. ZNS-Infektion ohne Hautbeteiligung wird die VZV-PCR aus Liquor durchgeführt. Bei Verdacht auf okularen Zoster kann VZV-DNA im Kammerwasser und z.T. auch in Augenabstrichen nachgewiesen werden. Liegt der Verdacht auf eine systemische Infektion mit Organbeteiligung vor, muss VZV-DNA im Serum oder Plasma nachgewiesen werden. Dabei sollte eine quantitative PCR durchgeführt werden, da geringe Mengen an VZV-DNA häufig auch beim lokalisierten Zoster im Serum oder Plasma nachweisbar sind (Tabelle 1 (Tab. 1)) [37].

### 4.3 Antigennachweis

Der direkte Antigennachweis, bei dem Proteine des VZV mittels monoklonaler, fluoreszenzmarkierter Antikörper in Abstrichen nachgewiesen werden, ist im Vergleich zu den molekularbiologischen Verfahren deutlich weniger sensitiv und spezifisch und kann daher nicht mehr empfohlen werden [32], [33], [38], [39]. Auch kann der Antigennachweis nur aus flüssigkeitsgefüllten Bläschen in den ersten Tagen der Erkrankung gelingen. Der früher übliche Tzanck-Test (histologischer Nachweis intranukleärer Einschlusskörperchen) kann als Kliniktest bei der Abgrenzung nicht-herpesviraler Effloreszenzen nützlich sein, auch wenn Sensitivität und Spezifität gegenüber den virologischen Methoden deutlich niedriger sind. Zum Einsatz der Immunhistochemie siehe Kapitel 4.6 [40]. 

### 4.4 Antikörperdiagnostik

Die Serologie zur Bestimmung der VZV-spezifischen IgM-, IgG- und IgA-Antikörper mittels Immunoassay ist für die Akutdiagnostik der Zostereffloreszenzen nicht geeignet, kann aber bei der Abklärung zosterähnlicher Schmerzen oder zosterassoziierter (Facialis-) Paresen hilfreich sein. Im Verlauf der Erkrankung steigen die Konzentrationen der IgG-Antikörper sehr deutlich an, zusätzlich können häufig IgA-Antikörper und seltener auch IgM-Antikörper gegen VZV nachgewiesen werden. Die Messwerte sollten im Vergleich zu einem Ausgangsserum bewertet werden, da subklinische Reaktivierungen ebenfalls zu einem Anstieg der Antikörpertiter führen können. Damit ist ein Einzelserum für die Diagnostik nur dann geeignet, wenn ein auffällig hoher Messwert nach Einschätzung des jeweiligen Labors vorliegt [41], [42]. Zu beachten ist auch, dass ein serologischer Verlauf immer mit derselben Labormethode, idealerweise in Parallelmessung beurteilt werden muss. 

Der VZV-Antikörpernachweis im Liquor kann (unter Berücksichtigung einer Blut-Hirnschrankenstörung) nützlich sein, wenn der frühzeitige VZV-DNA-Nachweis verpasst worden ist. Dies gilt ebenfalls für den intraokularen VZV-Nachweis im Auge aus Kammerwasser und ggf. Glaskörper [43], [44].

### 4.5 Viruskultur

Kulturen aus humanen diploiden Lungenfibroblasten (WI-38 oder MRC-5) oder aus humanen retinalen Pigmentepihtelzellen (RPE) erlauben eine Virusisolation. Entsprechend dem zweiten Koch-Henle-Postulat wurde sie lange Zeit als Goldstandard gesehen. Aufgrund der Instabilität des stark zellassoziierten Herpesvirus schwankt die Sensitivität zwischen 20% und unter optimalen Bedingungen 80% [32], [39], [45], [46], [47]. RPE-Zellkulturen erbringen eine deutlich verbesserte VZV-Produktion [48]. VZV-induzierte zytopathische Effekte erscheinen in der Regel nach drei bis acht Tagen (Mittel: 7,5 Tage) [46]. Shell-Vial-Kulturen ermöglichen den Nachweis spezifischer Virusantigene vor dem Auftreten zytopathischer Effekte [49]. Viruskulturen stellen eine nützliche Möglichkeit dar, wenn Replikations-kompetente Virusisolate zur Testung der Medikamentensensitivität oder zur molekularen Charakterisierung benötigt werden (siehe Anmerkung unter 4.2). Die Virusisolierung in Zellkultur hat aufgrund ihrer niedrigen Sensitivität und dem höheren labordiagnostischen Aufwand ihren Stellenwert nur noch für besondere Fragestellungen.

### 4.6 Besondere Situationen

- **Zoster ophthalmicus**, insbesondere der Befall der nasociliaren Teilung des Nervus ophthalmicus, sichtbar als Hutchinson-Zeichen in Form von Papulovesikeln seitlich der Nase sowie im Bereich der Nasenspitze, ist mit einer hohen Komplikationsrate verbunden. Der Zoster ophthalmicus betrifft ca. zehn bis zwanzig Prozent aller Zostermanifestationen. Etwa die Hälfte dieser Fälle weist mehr als nur eine Hautbeteiligung, am häufigsten Keratitis, Konjunktivitis und Uveitis auf [50], [51]. Bedeutende Komplikationen stellen die verzögerte Keratitis, Skleritis, Augenlidretraktion, okulomotorische Lähmungen, paralytische Ptosis, sekundäre intraokuläre Drucksteigerung, Optikusneuritis oder eine akute retinale Nekrose mit dem Risiko der beidseitigen Erblindung dar [43], [52]. Die Gesamtinzidenz für Zoster ophthalmicus wurde kürzlich (2014, USA) mit 30,9 pro 100.000 Personenjahre (95%-Konfidenzintervall (KI): 25,9–36,6) angegeben. Die Inzidenz stieg ab 65 Jahren auf 104,6 pro 100.000 Personenjahre (95%-KI: 79,0–135,9) [53]. Die Augenbeteiligung bei Zoster ophthalmicus kann mit einer zeitlichen Verzögerung von mehr als vier Wochen auftreten. Bei Zoster ophthalmicus kommt es in etwa 10% der Fälle zu einer Keratitis oder Uveitis, was mit einem erhöhten Risiko von Beeinträchtigungen des Sehvermögens einhergeht [52], [54]. Da eine (intra-) okuläre Beteiligung häufig ist und bei der allgemeinen Untersuchung nicht auffallen könnte, wird bei Gesichtsbefall im Rahmen einer Zoster-Erkrankung zur Anpassung des Therapieregimes und die Notwendigkeit augenärztlicher Verlaufskontrollen eine augenärztliche Mitbehandlung empfohlen (Tabelle 2 (Tab. 2)). Die genaueste Methode zur Sicherung einer intraokulären Beteiligung ist der Nachweis von VZV-DNA [55], [56].
- **Zoster oticus:** Beim Zoster oticus breitet sich die Infektion im Gebiet der Hirnnerven VII und VIII aus. Die Diagnose wird in der Regel klinisch gestellt. Bei fraglicher viraler Ätiologie ist der Virusnachweis zu führen. Es sollten befundbezogen spezifische neuro-otologische Untersuchungen wie gegebenenfalls Tonaudiogramm, Sprachaudiogramm, Stapediusreflexprüfungen, otoakustische Emissionen (TEOAE), Hirnstammaudiometrie (BERA) und Vestibularisprüfungen (Koordinationsprüfungen, Videonystagmografie, kalorische Erregbarkeitsprüfungen, Video-Kopf-Impuls-Test, vestibulär evozierte myogene Potentiale) erfolgen. Klinisch charakteristische Zeichen sind Ohrenschmerzen, Hörminderung bis Hörverlust (Schallempfindungsschwerhörigkeit), Schwindel, Gesichtsnervenlähmung und vesiculäre Effloreszenzen auf der Ohrmuschel und im äußeren Gehörgang [57]. Das Ramsay-Hunt-Syndrom umfasst einen Zoster oticus in Verbindung mit einer peripheren N.-facialis-Parese und möglicher Beeinträchtigung weiterer Hirnnerven wie manchmal auch V, IX und X [58], [59], [60]. Durch den Befall motorischer, sensibler und sensorischer Nervenfasern können Störungen der Gesichtsmuskulatur, des Hörens und des Gleichgewichtes, Sensibilitätsausfälle und Störungen der Schmeckfunktion, der Tränen-, Nasen- und Speichelsekretion auftreten [58], [61], [62], [63]. Durch individuelle Anastomosen zwischen Hirn- und Zervikalnerven variieren die dermatologischen Befunde. Aufgrund des erhöhten Risikos schwerer Komplikationen [63] wird insbesondere bei Befall des N. facialis und/oder des N. vestibulocochlearis eine Mitbehandlung durch den Hals-Nasen-Ohren-Arzt und Neurologen empfohlen, um eine Therapieanpassung und Hals-Nasen-Ohren-ärztliche bzw. neurologische Verlaufskontrollen festzulegen (Tabelle 3 (Tab. 3)) [60].
- **Zoster sine herpete** ist definiert als unilateraler dermatomaler Schmerz ohne kutane Läsionen bei Patienten mit virologischem und/oder serologischem Nachweis einer VZV-Infektion. Die genaueste Methode zur Diagnosesicherung ist der Nachweis ansteigender anti-VZV-IgG und -IgM. Die Bestimmung spezifischen Serum-IgAs kann ergänzend Aufschluss geben, da dies bei akuter Infektion neben IgM häufig und teilweise früher erhöht ist [59], [64], [65]. Bei Zoster sine herpete mit Fazialislähmung kann der VZV-DNA-Nachweis durch einen Nasen-Rachen-Abstrich zwei bis vier Tage nach Beginn der Fazialislähmung oder direkt aus dem Plasma mittels PCR erfolgen [66] (Tabelle 4 (Tab. 4)).
- **Atypische kutane Manifestationen** des Zoster werden als verruköser [67], lichenoider [68], follikulärer [69], [70], granulomatöser [71] Zoster und granulomatöse Angiitis [72], [73], [74], [75], [76] beschrieben. Bei atypischen kutanen Manifestationen wird eine diagnostische Hautbiopsie zum Nachweis des Virus mittels Immunhistochemie, in situ-Hybridisierung oder PCR am Nativpräparat empfohlen [77]. Hier kann auch eine begleitende serologische Untersuchung auf das Vorliegen einer aktiven VZV-Infektion nützlich sein. Bei Ulzerationen oder Nässen atypischer kutaner Manifestationen kann ein Abstrich zur Antigendetektion oder besser zum PCR-Nachweis erfolgen (Tabelle 5 (Tab. 5)) [40].
- **Zoster bei Kindern** ist der Erkrankung im Erwachsenenalter ähnlich. Die Schmerzsymptomatik ist in der Regel weniger stark ausgeprägt [78], [79], [80].
- **Rezidivierender Zoster** ist bei immunkompetenten Patienten ungewöhnlich und wurde in einer Periode von acht Jahren bei 6,2% der Patienten, bei Immunsuppression in 30% der Krankheitsfälle beobachtet. Die Daten zu Fällen zu rezidivierendem Zoster divergieren [81], [82], [83], [84], [85].

### 4.7 Komplizierte Verläufe des Zoster 

#### 4.7.1 Postzosterische Neuralgie

Die häufigste chronische Folgeerscheinung des akuten Zoster stellt die postzosterische Neuralgie dar, die gewöhnlich als Schmerz mit Persistenz von mehr als drei Monaten oder länger nach Abheilung der Hautläsionen definiert wird. Die Inzidenz und der Schweregrad der postzosterischen Neuralgie korreliert mit dem Patientenalter, mit steigender Häufigkeit jenseits des 50. Lebensjahres [13], [86], [87]. Patienten mit Zoster ophthalmicus mit Keratitis oder intraokulärer Entzündung haben ein höheres Risiko für eine postzosterische Neuralgie [86]. Folgende Risikofaktoren zur Bestimmung des individuellen Risikos für eine postzosterische Neuralgie werden vorgeschlagen: weibliches Geschlecht, Alter >50 Jahre, Anzahl der Läsionen >50, kraniale/sakrale Lokalisation, hämorrhagische Läsionen sowie prodromaler Schmerz [88]. In den meisten Fällen verbessert sich die postzosterische Neuralgie stetig. 

#### 4.7.2 Disseminierter Zoster und neurologische Komplikationen

Der Verlauf des Zoster ist bei einem disseminierten Hautbefund und/oder konfluierenden Läsionen schwerwiegender und ausgeprägter. Das Spektrum reicht dabei von einzelnen Organbeteiligungen und kann mit einer guten Prognose bis zum Multiorganversagen, dem sogenannten viszeralen Zoster reichen, der trotz hochdosierter intravenöser antiviraler Systemtherapie häufig fatal verlaufen kann [89], [90]. 

Patienten mit Risiko eines schweren Zoster und erhöhtem Risiko für eine kutane und/oder systemische Dissemination wie auch einer schweren postzosterischen Neuralgie können durch einige Risikofaktoren wie das Alter >50 Jahre [13], [86], [91] moderater bis schwerer prodromaler oder akuter Schmerzen [86], Immunsuppression [13], [91], [92], [93] inkl. Tumoren, Hämopathien, HIV-Infektion, Organ- oder Stammzelltransplantation sowie andere immunsuppressive medikamentöse Therapien identifiziert werden. Einige klinische Befunde zu Beginn der Zostererkrankung können auf ein erhöhtes Risiko für Komplikationen hinweisen: Satellitenläsionen (aberrierende Bläschen) [94], schwerer Ausschlag und/oder Befall mehrerer Dermatome oder ein multisegmentaler Zoster [95] sowie simultanes Auftreten von Läsionen unterschiedlicher Entwicklungsstadien, reduzierter Allgemeinzustand, meningeale oder andere neurologische Zeichen und Symptome. Die Expertengruppe empfiehlt gezielte klinische Untersuchungen auf genannte Symptome bei Patienten mit Zoster (Tabelle 6 (Tab. 6)). Tabelle 7 (Tab. 7) gibt einen Überblick über Risikofaktoren für einen komplizierten Verlauf.

Bei Patienten mit Zoster der Kopf-Hals-Region ist ein asymptomatischer Befall des ZNS häufig [96]. Selbst bei asymptomatischen Verläufen findet sich bereits bei bis zu 60% der Patienten ein pathologischer Liquorbefund [96]. Neben anderen Symptomen wird über eine Enzephalitis, Meningoenzephalitis, Myelitis, Cerebellitis, cerebrovaskuläre Erkrankungen inklusive Zoster-assoziierter Vaskulitis/Vaskulopathie, Radikulitis und das Guillan-Barré-Syndrom als Zoster-assoziiert berichtet, insbesondere bei immunsupprimierten und älteren Patienten [70], [82], [83], [84]. Unter Vaskulopathie wird die VZV-Infektion zerebraler Arterien verstanden, die zu ischämischem und hämorrhagischem Schlaganfall führen kann. Das Schlaganfallrisiko ist innerhalb eines Jahres nach Zoster um 30% erhöht [9]. Bei Zoster im Bereich des ersten Astes des Nervus trigeminus ist mit einem 4,5-fachen Schlaganfallrisiko zu rechnen [76]. Eine ganz neue Entwicklung kann der Nachweis von VZV-Antigenen, VZV-DNA sowie VZV-Viruspartikeln in den Temporalarterien von Patienten mit Riesenzellarteriitis zur Folge haben [73]. Die Bestätigung dieses Ergebnisses könnte möglicherweise in Zukunft die additive Behandlung von betroffenen Patienten mit Kortiokosteroiden und systemischen Nukleosidanaloga wie Aciclovir bedeuten [97].

Neurologische Komplikationen des Zoster sind gerade bei älteren Patienten nicht selten. Insbesondere durch eine zunächst subklinische virale Inflammation im Liquor und ZNS können Verwirrtheitszustände bei multimorbiden Patienten auftreten. Bei immunsupprimierten oder älteren Patienten >80 Jahre wird unabhängig von neurologischen Symptomen eine neurologische Untersuchung empfohlen (Tabelle 8 (Tab. 8)). Im Falle akuter fokaler neurologischer Dysfunktionen oder anderer neurologischer Zeichen und Symptome bei Patienten mit Zoster sollte eine neurologische Vorstellung/Mitbehandlung erfolgen (Tabelle 9 (Tab. 9)). Bei Verdacht auf Enzephalitis, Myelitis oder Zeichen eines akuten Schlaganfalls sollte eine kraniale oder spinale Magnetresonanz-Tomographie (MRT) mit MR-Angiographie der hirnversorgenden Gefäße durchgeführt werden.

Eine Enzephalitis oder Meningoenzephalitis ist eine seltene Komplikation, die sich bei etwa 0,25% der Patienten mit einem Zoster nachweisen lässt. Das typische klinische Bild ist im Vergleich zur Erstinfektion mit VZV- oder der HSV-Enzephalitis zumeist mild mit leichten Bewusstseinsstörungen, Verwirrtheit insbesondere bei Patienten >80 Jahre sowie Kopfschmerzen, leichter Nackensteife und Fieber bei einer meningitischen Mitbeteiligung. Neurologische Fokalsymptome wie eine Hemiparese oder Hirnstammsymptome und Krampfanfälle sind selten und zumeist Hinweis auf eine Vaskulopathie als weitere mögliche Komplikation. Bei Verdacht auf eine Enzephalitis sollte umgehend eine Bildgebung (präferentiell cMRT) und eine Lumbalpunktion inklusive VZV-PCR erfolgen. Bei einer Enzephalitis ist bis zu einer beginnenden Besserung der Symptome eine Monitorüberwachung auf einer Intermediate Care oder Intensivstation notwendig. Patienten mit einer Enzephalitis benötigen zudem häufig eine Anschlussheilbehandlung zur Rehabilitation v. a. der kognitiven Defizite. Eine reine Meningitis kann ebenfalls zu kognitiven Defiziten beitragen, auch wenn dies deutlich seltener ist als bei einer bakteriellen Meningitis [98]. Die Therapie einer Enzephalitis bzw. Meningitis sollte über 10–14 Tage mit intravenösem Aciclovir erfolgen. Eine zusätzliche Kortisongabe ist nicht sinnvoll, da es keine Studien gibt, die eine Wirksamkeit belegen. 

Es konnte gezeigt werden, dass Zoster einen unabhängigen Risikofaktor für vaskuläre Erkrankungen, besonders für Schlaganfälle, transiente ischämische Attacken und Myokardinfarkte darstellt [88], [89], [90]. Deshalb wird empfohlen, besondere Aufmerksamkeit auf akute kardiale und cerebrovaskuläre Symptome zu legen (Tabelle 10 (Tab. 10)). Neuere Studien deuten dabei auf eine Vaskulopathie bzw. Vaskulitis durch die VZV-Reaktivierung hin. Dabei können sowohl die großen als auch die mittleren und kleinen Arterien betroffen sein. Patienten mit einer zosterassoziierten Vaskulitis/Vaskulopathie können dabei ohne vorherige Anzeichen einer ZNS-Beteiligung sowohl plötzlich auftretende fokalneurologische Defizite wie eine Hemiparese als auch unspezifische Symptome wie Verwirrtheit, kognitive Defizite und Krampfanfälle aufweisen [99]. Bei V. a. eine VZV-Vaskulopathie sollte ein kraniales MRT inklusive MR-Angiographie ggf. mit Vessel wall imaging erfolgen. Eine Monitorüberwachung auf einer Intermediate Care/Stroke Unit oder Intensivstation ist zumindest in den ersten 24 Stunden notwendig. Je nach klinischem Befund und Besserung sollte eine Anschlussheilbehandlung zur neurologischen Rehabilitation erfolgen. Bei einer zosterbedingten Vaskulitis sollte neben der intravenösen Behandlung mit Aciclovir die Gabe von Kortison (mindestens 1 mg/kg KG bis zur Besserung) erfolgen. 

Eine systemische VZV-Disseminierung bei immunsupprimierten Patienten mit Zoster stellt die schlimmste akute Komplikation dar, die glücklicherweise selten ist. Hier wird empfohlen, dass Kliniker mögliche assoziierte Komplikationen wie eine Pneumonie, Hepatitis, disseminierte intravasale Koagulation und ZNS-Zeichen bei Patienten mit Zoster und akuter Allgemeinzustandsverschlechterung ausschließen (Tabelle 9 (Tab. 9)). 

### 4.8 Suche nach (versteckten) Risikofaktoren

Zoster wird als Indikator einer HIV-Infektion angesehen. In verschiedenen Darlegungen konnte eine erhöhte Prävalenz für HIV-Positivität bei Zoster-Patienten gezeigt werden, insbesondere bei Befall mehrerer Dermatome oder in Fällen von rezidivierendem Zoster sowie bei Nachweis weiterer Risikofaktoren für eine HIV-Erkrankung [100], [101], [102], [103]. Bei jüngeren Patienten (unter 50 Jahren) mit Zoster, insbesondere bei ausgeprägtem Befund mit multidermatomalem Befall oder rezidivierendem Zoster, Läsionen in unterschiedlichen Stadien oder bei weiteren Risikofaktoren für eine HIV-Erkrankung wird ein HIV-Suchtest empfohlen (Tabelle 11 (Tab. 11)). 

Eine Tumorsuche aufgrund einer Zoster-Infektion bleibt weiterhin Gegenstand von Debatten. In einer großen Kohorte von Zoster-Patienten wurden die Inzidenzraten verschiedener Tumoren untersucht. In der vorliegenden Analyse gab es keine standardisierte Erhöhung der Inzidenzraten [104]. Im Gegensatz dazu zeigte eine retrospektive kontrollierte Kohortenstudie eine Hazard Ratio (HR) von 2,43 (95% Konfidenzinterval 2,21–2,66) für das Risiko, nach der Zoster-Erkrankung an einem Tumor zu erkranken [105]. Basierend auf diesen widersprüchlichen Daten und klinischem Konsens empfiehlt die Expertengruppe keine Tumorsuche, die allein auf der Zoster-Erkrankung beruht (Tabelle 12 (Tab. 12)). 

### 4.9 Andere spezifische Situationen

Resistenzen von VZV-Infektionen auf Aciclovir wurden als fehlendes klinisches Ansprechen oder Virusnachweis unter antiviraler Systemtherapie nach 10–21 Tagen definiert [106], [107]. Dies wurde insbesondere bei VZV-Infektion immundefizienter Patienten vor allem nach hämatopoetischer Stammzelltransplantation beschrieben [67], [108], [109], [110].

Die phänotypische Bestimmung der Aciclovirresistenz in vitro wird als Goldstandard der Resistenztestung angesehen. Allerdings ist die in vitro-Testung nur begrenzt verfügbar und hat aufgrund der VZV-Isolation aus der Zellkultur eine niedrige Sensitivität. Die Genotypisierung des VZV erfolgt schneller und kann ebenfalls Aufschluss über Aciclovir-resistente VZV während einer Langzeitbehandlung geben. 

Im Gegensatz zu HSV [111] sind die natürlichen und Aciclovirresistenz-assoziierten Polymorphismen der Thymidinkinase (TK) und Polymerase bei VZV noch unvollständig definiert und erlauben deshalb für diagnostische Zwecke noch keine komplette Aussagefähigkeit [107], [111], [112], [113]. Die VZV-Genotypisierung wird nur in spezialisierten Laboren durchgeführt. 

Ein Zoster kann auch durch die Reaktivierung der Impfviren verursacht werden, die nach der Varizellenimpfung in den Neuronen persistieren. Die Unterscheidung zwischen Wild- und Impftypviren ist durch die Genotypisierung der viralen DNA möglich [114], [115]. 

### 4.10 Hygienemaßnahmen im Krankenhaus

Immunkompetente Zoster-Patienten scheiden in aller Regel über den Oropharynx keine infektiösen Viruspartikel aus, so dass auch keine Übertragung durch Tröpfchen oder Aerosole stattfinden kann [116]. 

Dass in einigen der wenigen durchgeführten Untersuchungen sowohl bei Gesunden als auch bei Zosterpatienten vermehrungsfähiges VZV im Speichel nachgewiesen werden konnte, ist wegen der niedrigen Infektionsdosis für die Kontagiosität belanglos. Dies wird von der Kommission für Krankenhaushygiene und Infektionsprävention (KRINKO) auch so bewertet, indem ausschließlich von einer Virusübertragung durch direkten oder indirekten Kontakt zu den Zosterläsionen ausgegangen wird, die bis zur vollständigen Verkrustung der Bläschen infektiös sind. Aus verkrusteten Läsionen lässt sich in der Regel kein Virus anzüchten.

Der Ratgeber des Robert Koch-Instituts (RKI-Ratgeber) „Varizellen und Herpes zoster“ führt dazu aus: „Bei strenger Einhaltung der Basishygiene und bei kooperativen Patienten kann durch eine vollständige Abdeckung der Läsionen die Übertragungswahrscheinlichkeit reduziert werden. Die KRINKO empfiehlt auch für Patienten mit Zoster eine Isolierung im Einzelzimmer bis zur vollständigen Verkrustung aller Läsionen. Wenn eine Einzelzimmerisolierung nicht möglich ist, kann nach individueller Risikoabwägung eine gemeinsame Unterbringung mit Patienten mit dokumentierter Immunität gegen VZV erwogen werden [117]. Schutzkleidung muss in diesen Fällen nicht zwingend schon bei Betreten des Zimmers, aber bei Verrichtungen am Patienten mit noch nicht verkrusteten Blasen getragen werden. Wesentlich ist dabei der Ausschluss einer Übertragung auf immunkompromittierte Kontaktpatienten (Tabelle 13 (Tab. 13)).

Bei immunsupprimierten Patienten mit disseminiertem Zoster hält die Expertengruppe eine Ausbreitung über Aerosole für möglich und würde wie bei Varizellen verfahren.

## 5 Therapie

### 5.1 Antivirale Medikation 

#### 5.1.1 Generelle Aspekte zur antiviralen Medikation

Der Zoster verläuft bei Patienten ohne Risikofaktoren für Komplikationen in der Regel selbstlimitierend. Therapieziele sind die Verbesserung des Outcomes in Bezug auf die Lebensqualität (QoL) der betroffenen Patienten, Dauer und Ausbreitung der kutanen Symptome sowie die Intensität und Dauer des akuten Zoster-assoziierten Schmerzes. Ein wichtiges sekundäres Behandlungsziel ist die Reduktion der Inzidenz der postzosterischen Neuralgie, der häufigsten Folgeerkrankung des Zoster. Bei immunsupprimierten Patienten oder anderweitig anfälligen Patienten werden die Behandlungsziele um Reduktion der Inzidenz und Intensität von begleitenden Komplikationen erweitert [118], [119].

In kontrollierten Studien konnte eine verkürzte Dauer der Hautläsionen sowie Dauer oder Schwere des Zoster-assoziierten Schmerzes durch systemische Behandlung mit Aciclovir [120], [121], [122], [123] und Famciclovir [124] im Vergleich zu Placebo gezeigt werden. Eine Metaanalyse von vier Placebo-kontrollierten Studien fand eine statisch signifikante Überlegenheit von oralem Aciclovir gegenüber Placebo in Hinblick auf die Zeit bis zur Beendigung des Schmerzes [125]. Ergebnisse aus randomisiert-kontrollierten Studien deuten auf die Überlegenheit von Valaciclovir gegenüber Aciclovir zur Behandlung der Dauer und/oder Schwere des Zoster-assoziierten Schmerzes hin [126], [127]. In diesen Studien wurde kein statistisch signifikanter Unterschied für die Dauer der Hautläsionen nachgewiesen. Ergebnisse aus randomisierten kontrollierten Studien, die Famciclovir mit Aciclovir [128], [129], Brivudin mit Aciclovir [130] sowie Valaciclovir mit Famcicolvir [131] verglichen, ergaben keine statistisch signifikanten Unterschiede für die Dauer des Schmerzes und Dauer der Hautläsionen. In einer weiteren randomisiert kontrollierten Studie war Famciclovir in Hinblick auf die Dauer des Schmerzes Aciclovir überlegen, wenn die Famciclovirdosis verdoppelt wurde. [132] Eine andere randomisiert-kontrollierte Studie mit Valaciclovir vs. Famciclovir zeigte eine statistisch signifikante frühere Schmerzreduktion durch Famciclovir [133].

Lebensqualität (QoL) als zentrales patient-reported outcome (PRO) wurde nur in wenigen klinischen Studien untersucht. Aufgrund der Reduktion der Dauer und Intensität des akuten Zoster-assoziierten Schmerzes wird angenommen, dass die antivirale Therapie die Lebensqualität der Patienten positiv beeinflusst. Diese Vermutung basiert jedoch nicht auf wissenschaftlichen Beobachtungen. 

In einem systematischen Review konnte gezeigt werden, dass weder Aciclovir noch Famciclovir im Placebovergleich die Inzidenz der postzosterischen Neuralgie vier bis sechs Monate nach akuter Zosterinfektion statistisch signifikant reduziert [134].

Brivudin wurde im Rahmen eines Fragebogens als Studien-Follow-up einer zuvor durchgeführten randomisierten kontrollierten Studie mit Aciclovir verglichen [130]. Eine signifikant niedrigere Inzidenz einer postzosterischen Neuralgie nach Brivudineinnahme als nach Aciclovirbehandlung wurde festgestellt [135]. In einer weiteren randomisierten kontrollierten Studie, die Brivudin mit Famciclovir verglich, konnte kein statistisch signifikanter Unterschied beider Gruppen hinsichtlich der Prävalenz und Dauer des Schmerzes nachgewiesen werden [136]. In weiteren randomisierten kontrollierten Studien konnten statistisch signifikante Unterschiede zwischen Valaciclovir und Aciclovir [137] sowie Famciclovir und Aciclovir [138] nicht nachgewiesen werden. 

Kontrollierte Studien der antiviralen Medikation wurden ebenfalls an immungeschwächten Patienten durchgeführt: Eine randomisiert-kontrollierte Studie verglich die Wirksamkeit von intravenösem Aciclovir mit Placebo bei immungeschwächten Patienten mit lokalisiertem oder disseminiertem Zoster. Eine Überlegenheit von Aciclovir hinsichtlich der Inzidenzreduktion von Komplikationen (einschließlich kutaner und viszeraler Dissemination) konnte nachgewiesen werden [139]. Eine weitere randomisierte kontrollierte Studie an 48 immunsupprimierten Patienten verglich die Gabe von intravenösem Aciclovir mit oraler Gabe von Brivudin. Ein statistisch signifikanter Unterschied hinsichtlich der kutanen oder viszeralen Dissemination wurde nicht festgestellt [140]. Im Vergleich zu Vidarabin war Aciclovir in Bezug auf die Prävention einer kutanen Dissemination, Schmerzdauer und Heilung der Hautläsionen statistisch signifikant überlegen [141].

Die heute empfohlene antivirale Standardtherapie bei Zoster umfasst die vier oral wirksamen Nukleosidanaloga Aciclovir, Valaciclovir, Famciclovir und Brivudin sowie Aciclovir zur parenteralen Therapie (Tabelle 14 (Tab. 14)). In Übereinstimmung mit vorherigen Leitlinien [6], [142] empfiehlt die Expertengruppe die Initiierung einer antiviralen Systemtherapie für die in Tabelle 15 (Tab. 15) aufgelisteten Patientengruppen (Empfehlung #20, Tabelle 15 (Tab. 15)). Aufgrund des relativ geringen Nebenwirkungsrisikos durch eine antivirale Medikation sollte eine antivirale Systemtherapie auch bei Patienten mit geringem Risiko für Folgeerscheinungen oder komplizierte Verläufe in Betracht gezogen werden (Empfehlung #21, Tabelle 15 (Tab. 15)). 

Bei Patienten mit kompliziertem Zoster oder Risiko eines komplizierten Verlaufs wird konsensusbasiert die intravenöse Gabe von Aciclovir empfohlen (Empfehlung #22, Tabelle 16 (Tab. 16)).

Faktoren, die für die Präferenz eines der oral anwendbaren antiviralen Medikamente entscheidend sind, wurden in Tabelle 17 (Tab. 17) (Empfehlung #23) erfasst. Die Evidenz für die Überlegenheit von Valaciclovir, Famciclovir und Brivudin gegenüber oralem Aciclovir hinsichtlich der unterschiedlichen Outcomes ist unsicher. Brivudin bietet den Vorteil einer reduzierten Einnahmefrequenz, ist aber nicht in allen Ländern verfügbar. Aciclovir verursacht die geringsten Kosten. Brivudin ist kontraindiziert bei immunsupprimierten Patienten sowie aufgrund möglicher lebensbedrohlicher Arzneimittelinteraktionen bei Patienten, die in den letzten vier Wochen mit 5-Fluoropyrimidin-haltigen Medikamenten (z.B. 5-Fluorouracil, Flucytosin) behandelt wurden. 

Laut Fachinformation der jeweiligen Medikamente sind nierenwertbedingte Dosisanpassungen bei der Gabe von Aciclovir, Valaciclovir und Famciclovir notwendig. Bei Anwendung der genannten Medikamente sollte bei bekannter oder vermuteter Niereninsuffizienz eine Kreatininkontrolle zum Zeitpunkt des Behandlungsbeginns erfolgen (Tabelle 18 (Tab. 18)). 

Wegen fehlender Studiendaten für die Einleitung der antiviralen Therapie mehr als 72 Stunden nach Beginn der Hautläsionen gibt es keine Evidenz für eine diesbezügliche Empfehlung. Konsensusbasiert sowie bestehenden Leitlinien [6], [142] folgend, wird hier die Einleitung einer antiviralen Medikation zu einem späteren Zeitpunkt entsprechend der Auflistung in Empfehlung #25 (Tabelle 19 (Tab. 19)) empfohlen, wenn innerhalb der ersten 72 Stunden nach Beginn der Hautläsionen keine systemische Therapie eingeleitet werden konnte. 

Studien, in denen die antivirale Therapie über sieben Tage hinaus fortgesetzt wurde, ergaben keinen klinisch relevanten Unterschied [126] oder Zusatznutzen im Vergleich zur Standardtherapie [143]. Die Dauer der antiviralen Medikation sollte verlängert werden, bis keine vesikulären Läsionen mehr auftreten. Hält die Vesikelbildung länger als sieben Tage an, sollte die Diagnose neu bewertet und eine Resistenz des Virus auf die antivirale Medikation in Betracht gezogen werden.

### 5.1.2 Besondere Situationen

- **Niereninsuffizienz:** Bei niereninsuffizienten Patienten mit Zoster empfehlen wir bei Indikation zur oralen antiviralen Therapie Brivudin bzw. bei Indikation zur intravenösen Therapie eine nierenadaptierte Gabe von intravenösem Aciclovir (Tabelle 20 (Tab. 20)). Die Empfehlung erfolgt konsensusbasiert durch die Expertengruppe sowie auf der Tatsache, dass Brivudin geringer als andere antivirale Systemtherapeutika abhängig von der renalen Exkretion ist. Die Therapie stationärer Patienten mit Aciclovir erlaubt enge Nierenfunktionskontrollen während der Behandlung.
- **Zoster ophthalmicus:** Als Kritierien für eine ophthalmologische Konsultation wird vor allem die Manifestation im Versorgungsbereich des Nasoziliarnervs (Hutchinson-Zeichen) angesehen. Eine Beteiligung des Auges tritt bei bis zu 85% der Betroffenen auf. Trotz eines negativen Hutchinson-Zeichens kann das Auge betroffen sein. Sehminderung, Augenschmerzen, Photophobie und verminderte Hornhautsensibilität sind Indikatoren für die Beteiligung des Auges. Alle Patienten mit Zoster ophthalmicus/Zoster des 1. Trigeminusastes sollten umgehend Aciclovir intravenös (8–10 mg/kg KG über 7–10 Tage) erhalten und einem Augenarzt zum Ausschluss einer okulären Beteiligung vorgestellt werden. Die Therapiestrategie bei Zoster ophthalmicus sowie die Notwendigkeit für eine augenärztliche Nachkontrolluntersuchung sollte durch einen Ophthalmologen festgelegt werden. Die bisher erläuterten Therapieempfehlungen gelten prinzipiell ebenso im Falle eines Zoster ophthalmicus. Die akute retinale Nekrose als Komplikation eines Zoster ophthalmicus stellt einen ophthalmologischen Notfall dar und sollte unter enger augenärztlicher Überwachung behandelt werden. Da die akute retinale Nekrose rasch progredient ist und das kontralaterale Auge infizieren kann, ist eine sofortige intravenöse Induktionstherapie mit Fortführung einer oralen antiviralen Therapie für 3–4 Monate indiziert (Tabelle 21 (Tab. 21)). Die prologierte Behandlung wird zum Schutz des kontralateralen Auges empfohlen [144], [145]. Die zusätzliche Gabe von systemischen Glukokortikoiden bei Patienten mit akuter retinaler Nekrose wird in Hinblick auf die geeignete Einleitung kontrovers diskutiert. Eine Startdosis von 0,5–1,0 mg/kg KG Prednisolon am Tag für die ersten 7–10 Behandlungstage kann empfohlen werden [145], [146]. Wir empfehlen die Anwendung topischer und systemischer Glukokortikoide als ergänzende antiinflammatorische Therapie (Tabelle 21 (Tab. 21)). Vorsicht sollte bei Anwendung von Glukokortikoiden ohne parallele antivirale Therapie geboten sein, da dadurch die Virusreplikation gefördert werden und eine akute retinale Nekrose ausgelöst werden kann [147].
- **Zoster oticus:** Bei Zoster oticus mit Befall des N. facialis und/oder des N. vestibulocochlearis, Ohrenschmerzen und Schwindel sollte die Therapiestrategie durch einen Hals-Nasen-Ohren-Arzt und Neurologen festgelegt werden [60]. Die Expertengruppe empfiehlt eine Kombinationstherapie aus intravenösem Aciclovir und Glukokortikoiden (Tabelle 22 (Tab. 22)). Bei einem Zoster oticus mit ausgeprägten Schmerzen und Hirnnervenlähmungen wird eine antivirale Systemtherapie mit intravenösem Aciclovir gefolgt von oralem Aciclovir für weitere ein bis zwei Wochen erfolgreich angewendet [57], [148], [149]. Glukokortikoide werden weiterhin als die beste Behandlungsmöglichkeit bei viraler Entzündung des N. facialis angesehen [150]. Die Wirksamkeit der Glukokortikoidtherapie ergibt sich aus der Verminderung der entzündlich-ödematösen Schwellung und der daraus resultierenden Dekompression des N. facialis innerhalb des Canalis nervi facialis im Felsenbein [62], [151], [152]. Eine Kombinationstherapie ist effizienter hinsichtlich der Wiederherstellung der N.-facialis-Funktion nach Zoster oticus [153], [154], [155], [156] [157] und scheint eine bessere Prognose zu haben [158]. Hinsichtlich der Glukokortikoiddosierung wird auf die AWMF-Leitlinie „Idiopathische Fazialisparese (Bell’s Palsy)“, S2k-Leitlinie, Reg.Nr. 030-013 verwiesen [159]. Zusätzlich sind ausreichend Analgetika bei den meist ausgeprägten neuralgiformen Schmerzen und Antivertiginosa bei Schwindel indiziert. Kommt es trotz der Behandlung der Parese des N. facialis zu einer unvollständigen Ausheilung/Defektheilung des N. facialis, ist eine Vorstellung beim Neurologen und HNO-Arzt zu empfehlen.
- **Schwangerschaft:** Aufgrund fehlender systematisch erfasster Daten zur Sicherheit einer antiviralen Medikation während der Schwangerschaft wird ein vorsichtiger Einsatz unter Abwägung möglicher Schädigungen mit dem Therapienutzen empfohlen. Liegen keine Risikofaktoren für einen komplizierten Verlauf vor, wird bei Schwangeren mit Zoster eine systemische antivirale Therapie nicht empfohlen (Tabelle 23 (Tab. 23)). In einer großen Populations-basierten, retrospektiven, kontrollierten Kohortenstudie und in einer Studie, die Registerdaten beinhaltet, konnte gezeigt werden, dass bei Kindern von Müttern, die während der Schwangerschaft Aciclovir erhielten, kein erhöhtes Risiko für Fehlbildungen vorlag. Für andere antivirale Medikamente (Valaciclovir und Famciclovir) kann aufgrund zu geringer Fallzahlen kein Schluss gezogen werden [160], [161]. Daraus ergibt sich die Empfehlung, dass bei Risikofaktoren für einen komplizierten Verlauf bei Zoster in der Schwangerschaft Aciclovir empfohlen werden kann, wenn der potentielle Therapienutzen der Mutter das potentiale Risiko des Fetus überwiegt (Tabelle 23 (Tab. 23)).
- **Kinder:** Aufgrund fehlender Sicherheitsdaten der antiviralen Systemtherapeutika bei Anwendung im Kindesalter wird ein vorsichtiger Umgang mit Berücksichtigung möglicher Schädigung und Nutzen der antiviralen Therapie empfohlen. Im Allgemeinen geht ein Zoster im Kindesalter mit einer geringeren Morbidität als im Erwachsenenalter einher [78], [79]. Bei fehlenden Risikofaktoren wird keine antivirale Systemtherapie bei Kindern empfohlen (Tabelle 24 (Tab. 24)). Die Initiierung einer antiviralen Systemtherapie im Kindesalter kann bei Vorliegen von Risikofaktoren für einen komplizierten Verlauf (Tabelle 7 (Tab. 7)) erwogen werden, wenn der potentielle Therapienutzen das potentielle Therapierisiko überwiegt (Tabelle 24 (Tab. 24)).
- **Therapierefraktäre/chronische Zosterläsionen:** Klinisch kann bei fehlendem Therapieansprechen nach 10- bis 21-tägiger Aciclovir-Gabe von einer resistenten VZV-Infektion gesprochen werden [106], [107]. Dies tritt insbesondere bei verrukösen VZV-Infektionen auf [67]. Liegt eine Aciclovirresistenz vor, kann eine Therapieumstellung auf ein alternatives Virostatikum wie z.B. Brivudin oder ein anderes Thymidinkinase-abhängiges antivirales Medikament (Famciclovir) vorgenommen werden. In einer kleinen retrospektiven Fallserie mit immunsupprimierten Patienten mit Aciclovir-resistentem Zoster wurde ein Ansprechen auf intravenöse Gabe von Foscarnet beobachtet [106], [162]. Anekdotisch wird über Aciclovir-resistente VZV-Stämme berichtet, die auf Cidofovir ansprechen [163], [164], [165]. Beide Medikamente sind nicht für die Behandlung des Zoster zugelassen. Foscarnet und Cidofovir sollten aufgrund der möglichen schweren Nebenwirkungen nur in sehr schweren Fällen und in Rücksprache mit Virologen, Pharmazeuten und ausführlicher Diskussion der Risiko-Nutzen-Abwägung mit dem Patienten eingesetzt werden. Bei chronischen Zoster-Läsionen verweisen wir auf einen Review-Artikel von Wauters et al. [67] über chronische mukokutane Zosterläsionen.

### 5.2 Schmerzbehandlung

#### 5.2.1 Einleitung

Charakteristischerweise treten bei Zoster in den befallenen Dermatomen akute Schmerzen auf. Die Charakterisierung dieser Schmerzen ist entscheidend (Tabelle 25 (Tab. 25)). Einerseits handelt es sich um Schmerzen im Sinne von „Wundschmerzen“ (sog. nozizeptive Schmerzen), die im Rahmen der akuten Entzündungsreaktion entstehen. Andererseits führt die axonale Ausbreitung der VZV zu einer begleitenden Entzündung und damit ebenfalls zu Schmerzen (sog. akute Zosterneuralgie/neuropathischer Schmerz). Definitionsgemäß werden dermatomale Schmerzen, die länger als drei Monate nach Abheilung der Zosterläsionen an der Haut fortbestehen, als postzosterische Neuralgie (PZN) bezeichnet.

Akute Zoster-assoziierte Schmerzen treten bei >95% der Patienten im Alter von über 50 Jahren auf. In 60–70% der Fälle kommt es zu einem kontinuierlichen Schmerz mit Persistenz über einen Monat nach der Erkrankung, 40% der Patienten bezeichnen diesen Schmerz als schwer [166], [167].

#### 5.2.2 Erfassung der Schmerzintensität

Die Schmerzintensität sollte über eine validierte Bewertungsskala (z.B. Visuelle Analog Skala (VAS) oder Nummerische Rating Skala (NRS)) erhoben werden [168], [169] (Tabelle 26 (Tab. 26)). Ergänzend können validierte Bewertungsinstrumente zur Erfassung der neuropathischen Schmerzcharakteristika (Douleur Neuropathique 4 (DN4), PainDETECT (PD-Q) oder Leeds Assessment of Neuropathic Symptoms and Signs (LANSS)) [168], [169] sowie der Lebensqualität (SF36 oder als Kurzform SF12) [168], [169] herangezogen werden.

Weitere Instrumente können zur Erfassung des Therapieerfolges (z.B. minimaler und maximaler Schmerz in den letzten 24 Stunden, Schmerzintensität während der Bewegung, Zufriedenheit mit dem Schmerzmanagement (NRS: 0=unzufrieden, 10=sehr zufrieden; Tabelle 27 (Tab. 27)). Die Bewertungsinstrumente wurden kürzlich zur Erfassung des akuten postoperativen Schmerzes Europaweit validiert [170]. 

#### 5.2.3 Behandlung des Zoster-assoziierten Schmerzes

Während es reichlich Literatur über die postzosterische Neuralgie gibt [76], [139], liegt wenig Evidenz über die Behandlung des akuten Zoster-assoziierten Schmerzes vor.

Abgesehen von der Verbesserung des funktionellen Status und der gesundheitsbezogenen Lebensqualität dient die Kontrolle des akuten Zoster-assoziierten Schmerzes zur Risikoreduktion einer postzosterischen Neuralgie. Kontrollierte Studien existieren nicht, die diese Annahme belegen. Anders als bei der postzosterischen Neuralgie sollte der akute Zoster-assoziierte Schmerz bevorzugt mit systemischen Analgetika und nicht topisch behandelt werden (Tabelle 28 (Tab. 28)). Dabei sollte nicht vergessen werden, dass zum Teil die Neuroinflammation Ursache der schmerzhaften Empfindungen ist [171], [172].

**Abbildung 2:**
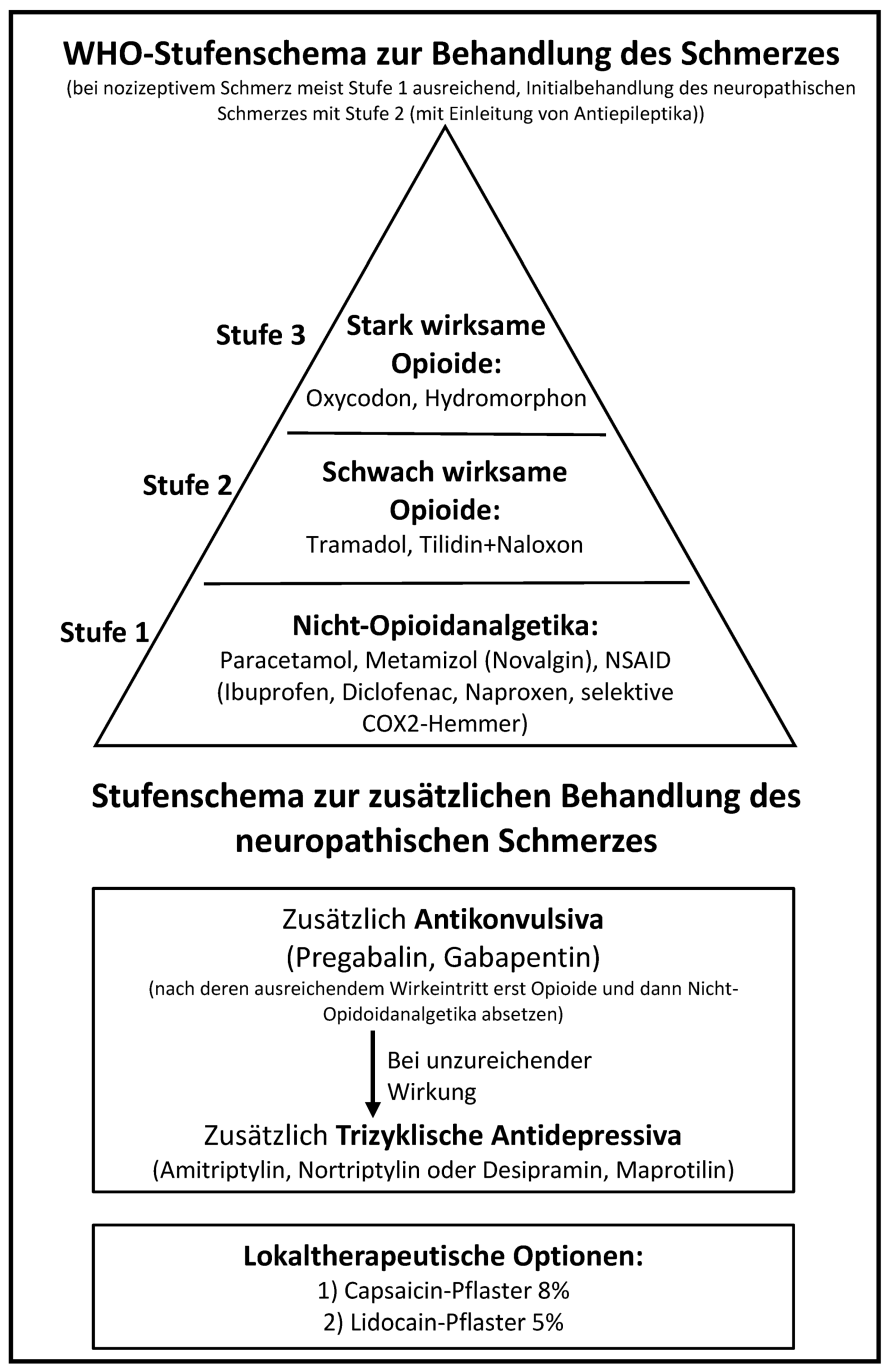
Abbildung 2: Schmerztherapie bei Zoster

Im Fall rein neuropathischer Schmerzen sollte wegen des verzögerten Ansprechens (Aufdosierphase) neben der Antiepileptikatherapie initial auch entsprechend WHO-Schmerzschema mit NSAR und Opioiden behandelt werden [173]. Nach dem Wirkeintritt der Antiepileptika sollte eine Reduktion bisheriger Analgetika (zunächst der Opioide, danach der Nicht-Opioidanalgetika) versucht werden (Abbildung 2 (Fig. 2)). 

Wenn Unsicherheit besteht, ob nur eine akute nozizeptive oder schon neuropathische Schmerzkomponente vorliegt, können Antiepileptika erwogen werden, da sie auch beim akuten Zosterschmerz eine moderate Überlegenheit gegenüber Placebo gezeigt haben (Tabelle 29 (Tab. 29), Tabelle 30 (Tab. 30)) [174]. 

Die Wirkung von sowohl Pregabalin als auch Gabapentin für den neuropathischen Schmerz bei Zoster ist in Studien und Metaanalysen belegt worden [175], [176], [177], [178]. Der Begriff postzosterische Neuralgie wird in den Studien nicht einheitlich verwendet und umfasst oft auch den Schmerz in der akuten Phase. Pregabalin ist schneller aufdosierbar und liegt in besser oral einnehmbarer Tablettenform vor. Eine effektive Plasmakonzentration der genannten Präparate wird nach einigen Tagen erreicht. Die Basisschmerztherapie durch Analgetika sollte daher nicht verzögert werden. 

Bis auf anfängliche Müdigkeit und Schwindel werden die Substanzen gut vertragen und schwere Medikamenteninteraktionen sind nicht bekannt. Unter Therapie mit Antiepileptika (Gabapentin, Pregabalin) sollte folgendes beachtet werden:

- sorgfältige Blutglukosekontrollen bei Diabetikern (bei einigen Patienten ist zu Beginn der Behandlung eine Insulinanpassung erforderlich)
- Kontrolle der Pankreasenzyme in der Aufdosierphase
- gegenseitige Beeinflussung von Gabapentin mit Opioiden in Bezug auf Wirkung, aber auch Nebenwirkung (Somnolenz, Sedierung, Atemdepression)
- schrittweises Absetzen von Gabapentin und Pregabalin (über sieben Tage; aufgrund des Risikos epileptischer Entzugsanfälle).

Antidepressiva blockieren die Wiederaufnahme der monoaminergen Transmitter Noradrenalin und/oder Serotonin im Rückenmark. Infolge der erhöhten Transmitterkonzentration wird die nozizeptive Transmission durch das Rückenmark gehemmt. Außerdem blockieren sie spannungsabhängige Natriumkanäle und haben indirekte sympatholytische Eigenschaften [179], [180], [181]. Amitriptylin, ein Serotonin- und Noradrenalin-Wiederaufnahmehemmer, ist zurzeit am besten untersucht und unterdrückt alle Schmerztypen: den brennenden Spontanschmerz, einschießende Schmerzattacken sowie evozierte Schmerzen. Die mittlere Dosis, die zur Schmerzreduktion notwendig ist, liegt unter der antidepressiven Dosis. Die Schmerzreduktion setzt erst nach einigen Tagen bis zwei Wochen ein [182]. Mundtrockenheit ist ein Parameter für die erreichte Zieldosis – zumindest bei der Behandlung der Depression. Andauernde Müdigkeit deutet auf eine zu hohe Dosierung hin. Die relativ selektiven Noradrenalin-Wiederaufnahmehemmer wie Desipramin haben weniger anticholinerge Nebenwirkungen und führen zu weniger Sedierung. Selektive Serotonin-Wiederaufnahmehemmer wie Fluoxetin und Paroxetin zeichnen sich durch ein günstigeres Nebenwirkungsspektrum aus. Leider zeigten die meisten kontrollierten Studien keinen oder nur einen geringen analgetischen Effekt. Wichtige unerwünschte Wirkungen sind die Entwicklung einer orthostatischen Hypotension aufgrund der sympatholytischen Eigenschaften sowie die durch Histamin-Rezeptorblockade verursachte Sedierung, außerdem Harnretention, Gedächtnisstörungen, Herzrhythmusstörungen und Mundtrockenheit (anticholinerge Wirkung). Kontraindikationen sind AV-Blockbilder, Herzinsuffizienz, Engwinkelglaukom, Pylorusstenose und Prostatahyperplasie. Vor der Behandlung sollte daher bei allen Patienten ein EKG abgeleitet werden. Wenn ein AV-Block I. Grades vorliegt, empfiehlt sich eine Rücksprache mit dem Kardiologen, zumindest aber wöchentliche EKG-Kontrollen (es gibt Fälle, in denen ein AV-Block Grad I innerhalb einer Woche in einen AV-Block Grad III überging oder Patienten gar wegen Asystolie schrittmacherpflichtig wurden). Wenn die eingesetzten Dosen über 100 mg/d liegen, empfehlen sich, insbesondere bei älteren Patienten, ebenfalls regelmäßige EKG- und Blutspiegelkontrollen. Außerdem besteht möglicherweise ein erhöhtes Blutungsrisiko bei Kombination mit Faktor-Xa-Inhibitoren (Apixaban), da sie indirekt auch die thrombininduzierte Thrombozytenaggregation hemmen und die Serotoninwiederaufnahmehemmung durch SSRI/SNRI am Thrombozyten ebenfalls aggregationshemmend wirkt.

Nach Abheilung der Bläschen und Erosionen ist Capsaicin eine weitere Option zur Behandlung des neuropathischen Schmerzes. Es ist ein Agonist des Vanilloid-Rezeptors auf den primär nozizeptiven Afferenzen. Es steht als Capsaicin-Pflaster (8%) zur Verfügung. Eine einmalige Applikation dieser Substanz führt zu einem massiven Einstrom von Calcium in die Zelle, einer damit verbundenen heftigen Erregung der Nozizeptoren und konsekutiv zu einem brennenden Spontanschmerz. Die chronische Applikation bewirkt eine Degradation und damit verbunden einen reversiblen Funktionsverlust der nozizeptiven Nervenendigungen in der Haut [183], [184]. Offen angewandte Zubereitungen mit 0,025–0,075% Capsaicin sind hingegen bei Patienten mit postzosterischer Neuralgie nur schwach wirksam. Die Creme muss 3–4 mal tgl. für 4–6 Wochen aufgetragen werden, damit sich eine Wirkung auf die Nozizeptoren entfalten kann (Capsaicin-Extrakt 1%, davon 2,5 bzw. 7,5 g auf 100 g Unguentum leniens) (Tabelle 30 (Tab. 30)) [185].

In einem 2017 aktualisierten Cochrane Review [186], [187] (zu neuropathischem Schmerz und zu sogenannter postzosterischer Neuralgie) wurde in allen Studien eine Linderung des Schmerzes berichtet, einhergehend mit besserem Schlaf und besserer Lebensqualität [186], [187]. 

Eine entscheidende Nebenwirkung ist ein heftiges Hautbrennen im Applikationsgebiet, das durch die anfängliche Reizung der Afferenzen entsteht. Viele Patienten brechen deshalb die Therapie frühzeitig ab, bevor das Capsaicin seine desensibilisierende Wirkung entfalten kann. Vorheriges Kühlen der Haut kann dieses Brenngefühl bei Applikation eines Capsaicin-Pflasters deutlich reduzieren [188]. Eine intensive Aufklärung über diese nur vorübergehend auftretende Nebenwirkung ist deshalb von entscheidender Bedeutung.

Lidocain-5%-Pflaster bei postzosterischer Neuralgie [189] oder neuropathischem Schmerz allgemein kann entsprechend Cochrane Reviews [186] mangels guter Studien nicht als Schmerzmittel der ersten Wahl empfohlen werden. Da die Studien aber eine Wirksamkeit in der Schmerzlinderung bescheinigen, empfiehlt die Expertengruppe es als zweite Wahl nach Capsaicin. 

Die Behandlung des akuten Zoster-assoziierten Schmerzes sollte eine optimale Schmerzbesserung bzw. mindestens eine Schmerzreduktion auf ein für den Patienten tolerierbares Level zum Ziel haben. Nachuntersuchungen bei Patienten mit akutem Zoster-assoziierten Schmerz werden empfohlen, die über die Abheilung der Hautläsionen hinausgehen. Bei persistierenden, für den Patienten nicht tolerierbaren Schmerzen sollte an einen Schmerzspezialisten verwiesen werden (Tabelle 31 (Tab. 31), Tabelle 32 (Tab. 32)). 

#### 5.2.3.1 Risikofaktoren für starke neuropathische Schmerzen bzw. postzosterische Neuralgie (PZN) 

Unten genannte Umstände sind häufiger mit neuropathischen Schmerzen oder postzosterischer Neuralgie (PZN) assoziiert. Bei Vorliegen kann eine prophylaktische (Schmerz-) Therapie erwogen werden [30], [190]. Das individuelle Risiko für eine postzosterische Neuralgie kann mit etlichen prognostischen Faktoren, vorgeschlagen von Meister et al. [88], geschätzt werden: 

Weibliches Geschlecht, Alter >50 Jahre, Anzahl der Läsionen >50, kraniale/sakrale Lokalisation, hämorrhagische Läsionen und dermatomaler Schmerz in der Prodromalphase. 

Weitere Untersuchungen nennen Alter >50 Jahre und Schmerzen von der Stärke ≥4/10 NRS [191] oder

- mäßigen bis schweren prodromalen oder akuten Schmerz,
- Immunsuppression (einschließlich Leukämien, HIV-Infektion, Malignom, Immunsuppression bei Stammzelltransplantation und anderen Transplantationen, z.T. auch Autoimmunerkrankungen; laut Forbes et al. [86] auch Raucher und Patienten mit Diabetes mellitus)
- ausgeprägten Hautbefall (z.B. >50 Bläschen) oder hämorrhagische Form [86], [192].

Antikonvulsiva wie Gabapentin in den angegebenen Dosen sind bei Risikofaktoren auch zur Prophylaxe gegeben worden. Die Ergebnisse der bisherigen, qualitativ nicht besonders hochwertigen Studien sind nicht einheitlich (positiv in einer unkontrollierten, offenen Studie in Kombination mit Valaciclovir bei akutem Zoster [191], nicht signifikant in einer prospektiven kontrollierten Studie (ebenfalls in Kombination mit Valaciclovir [193]); mindestens eine weitere Studie läuft zurzeit [194]. Die prophylaktische Gabe von Gabapentin kann bei akutem Zoster und Risikofaktoren für protopathischen Schmerz (Tabelle 33 (Tab. 33)) erwogen werden (z.B. Alter >50 Jahre und eine der anderen oben genannten Umstände), möglichst gleich zu Beginn (innerhalb der ersten drei Tage nach Erscheinen der Hauteffloreszenzen) und zusätzlich zur antiviralen Therapie.

#### 5.2.3.2 Therapieoptionen bei anhaltenden Schmerzen (postzosterische Neuralgie)

In einer küzlich erschienenen Metaanalyse konnte gezeigt werden, dass die Anwendung von Nervenblockaden – unter Verwendung von Lokalanästhetika und/oder Steroiden – bei Patienten, bei denen innerhalb von drei Wochen die Diagnose Zoster gestellt wurde, die Inzidenz einer postherpetischen Neuralgie nach 3, 6 und 12 Monaten signifikant gesenkt werden konnte [195] (neun Studien, 1.645 Patienten). In einer weiteren Subgruppen-Analyse zeigt sich, dass vor allem die epidurale und paravertebrale Nervenblockade sowie die wiederholte und nicht die Einzelblockade eine signifikante Senkung der Inzidenz von postherpetischer Neuralgie bewirkte [195]. 

Diese Ergebnisse werden unterstützt durch eine kürzliche retrospektive Untersuchung von 227 Fällen mit Zoster-assoziierten Schmerzen, in der sich zeigte, dass die Kombination aus systemischer Standardtherapie und epiduraler Nervenblockade sowohl bei akuten als auch bei chronischen Zoster-assoziierten Schmerzen eine bis zu fünffach höhere Wahrscheinlichkeit einer erfolgreichen Schmerzbehandlung und eine bis zu dreifach höhere Wahrscheinlichkeit einer vollständigen Remissison als nur die Standardtherapie allein hatte [196]. Jedoch sollte hinzugefügt werden, dass die epidurale Nervenblockade nur in die geübte Hand von Anästhesiologen und/oder Schmerzspezialisten gehört. 

### 5.3 Lokaltherapie

#### 5.3.1 Generelle Aspekte

Studien und somit eine ausreichende Evidenz zur Empfehlung einer Lokaltherapie bei akutem Zoster liegen nicht vor, außer für Zoster ophthalmicus (siehe dort) oder für die Lokaltherapie neuropathischer Schmerzen, die unter 5.2.3 behandelt wird. 

Eine klinisch relevante antivirale Wirkung der Lokaltherapie ist nicht zu erwarten, zumindest gibt es keine placebokontrollierten randomisierten Studien, welche die Wirksamkeit der Anwendung der Externa belegen (Tabelle 34 (Tab. 34)). Ziele der Lokaltherapie sollten daher sein, sofern überhaupt erreichbar:

1. Förderung der Heilung (zum Beispiel durch Aufweichen und Lösen der Krusten)
2. Verhinderung einer bakteriellen Infektion
3. subjektive Linderung im akuten Stadium sowie
4. gezielte Schmerztherapie (siehe unter 5.2.3)

Eine Lokaltherapie sollte stadiengerecht erfolgen. Im frischen Bläschenstadium kühlende, entzündungshemmende oder antiseptische Lösungen, bei verkrustenden Bläschen antiseptische und krustenlösende Gele.

Gute Erfahrung allgemein zur antiseptischen und krustenlösenden Wirkung von Polihexanid-haltigen Gelen (Hydrophiles Polihexanid Gel 0,04% oder 0,1% NRF 11.131) liegen vor, da die Feuchtigkeit der Gele die spaltende Aktivität der hauteigenen Proteasen fördert, so dass wir sie für verkrustende Läsionen des Zoster empfehlen (Expertenmeinung). 

Austrocknende oder adstringierende Effekte sind nicht förderlich für die Wundheilung, allenfalls mag die unbelegte Vermutung bestehen, dass durch Austrocknung bzw. durch Milieuwechsel von feucht nach trocken eine Infektion und damit Wundheilungsstörung verhindert wird. 

Topika, die eine solche Wirkung für sich veranschlagen und als lindernd empfunden werden, wirken wahrscheinlich eher über ihre kühlenden (sterile Kochsalzlösung 0,9%) oder zusätzlich antientzündlichen (z.B. Schwarzteeumschläge 15–20 Minuten, 6x tgl.) Eigenschaften. Sie können angewendet werden, so lange sie die Haut nicht zu sehr austrocknen. Die Anwendung adstringierender Zinkoxidlotion ist in einigen Zentren verbreitet, wird aber von uns kritisch gesehen, da das abdeckende Zink die klare Beurteilung der Effloreszenzen unmöglich macht und der kühlende Effekt auch anders erreicht werden kann.

Kühlend und antiseptisch wirken milde Antiseptika wie Polyhexanidlösung 0,02 oder 0,04% (NRF 11.128), Octenidinlösung (Octenidindihydrochlorid 0,1% in Basiscreme DAC oder alternativ in Anlehnung an NRF 11.145 mit Propylenglycol und Wasser, aber ohne Prednicarbat, Tabelle 33 (Tab. 33)) [197].

#### 5.3.2 Topische Therapie bei spezifischen Situationen

Die optimale Behandlungsstrategie bei Zoster ophthalmicus mit Augenbeteiligung bleibt kontrovers, da einige randomisiert-kontrollierte Studien widersprüchliche Ergebnisse gezeigt haben. Eine randomisiert-kontrollierte Studie, welche die Wirksamkeit von topischem Aciclovir gegenüber Betamethason bei Zoster-assoziierter Keratouveitis untersuchte, konnte zeigen, dass okuläre Symptome signifikant schneller rückläufig waren und Rezidive seltener in der Aciclovir-Gruppe auftraten [198]. In einer anderen Studie konnte in der Aciclovir-Gruppe eine verlängerte Abheilungszeit der okulären Entzündung im Vergleich zur Steroid-Gruppe gezeigt werden [199]. Konsensusbasiert empfiehlt die Expertengruppe die Anwendung von Aciclovir Augensalbe fünf Mal tgl. im betroffenen Auge (Tabelle 35 (Tab. 35)), insbesondere bei Fällen mit VZV-assoziierter Keratitis dendritica. 

Bei disciformer Keratitis, Endotheliitis und anteriorer Uveitis stellen topische Steroide (ggf. auch subkonjunktival appliziert) in Kombination mit der systemischen antiviralen Behandlung die Hauptsäule der Therapie dar (Tabelle 35 (Tab. 35)). Steroide sollten mit Vorsicht unter engmaschiger augenärztlicher Kontrollen angewandt werden, da es bei Fortschreiten der Erkrankung zu einer Verdünnung und sogar Perforation der Kornea, einem sekundären Glaukom und zur Superinfektion einer reaktivierten Keratitis dendritica kommen kann [200]. In Fällen mit epithelialer Beteiligung sollte auf die topische Steroidgabe verzichtet werden, da es sonst zu einer Dissemination der epithelialen Läsionen kommen kann. 

Bei Zoster oticus fehlt Evidenz aus Studien zur Empfehlung einer spezifischen topischen Therapie.

## Anmerkungen

### Autorenschaft

Gerd Gross und Lisa Eisert teilen sich die Erstautorenschaft.

### Interessenkonflikte

Umgang mit Interessenkonflikten siehe Tabelle 36 (Tab. 36).

## Figures and Tables

**Tabelle 1:**
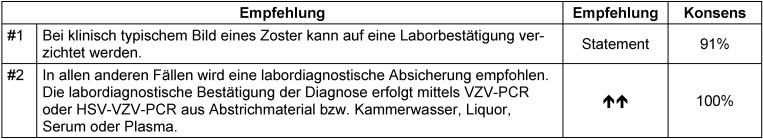
Tabelle 1: Diagnostische Empfehlungen zum Zoster, Empfehlung #1 und #2

**Tabelle 2:**
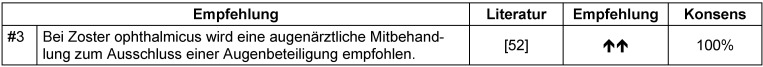
Tabelle 2: Diagnostische Empfehlungen zum Zoster, Empfehlung #3

**Tabelle 3:**
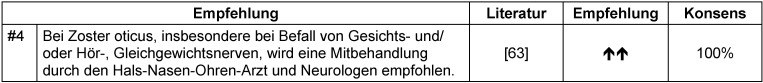
Tabelle 3: Diagnostische Empfehlungen zum Zoster; Empfehlung #4

**Tabelle 4:**
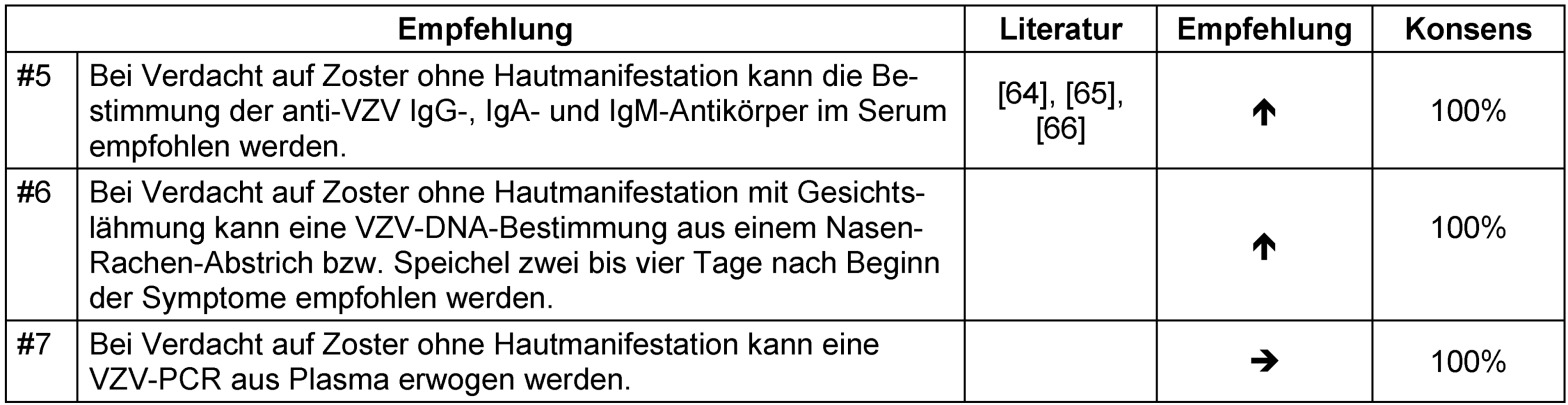
Tabelle 4: Diagnostische Empfehlungen bei Zoster; Empfehlung #5, #6 und #7

**Tabelle 5:**
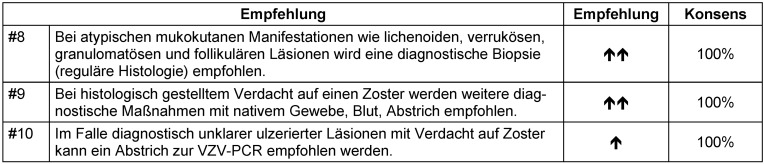
Tabelle 5: Diagnostische Empfehlungen bei Zoster; Empfehlung #8, #9 und #10

**Tabelle 6:**
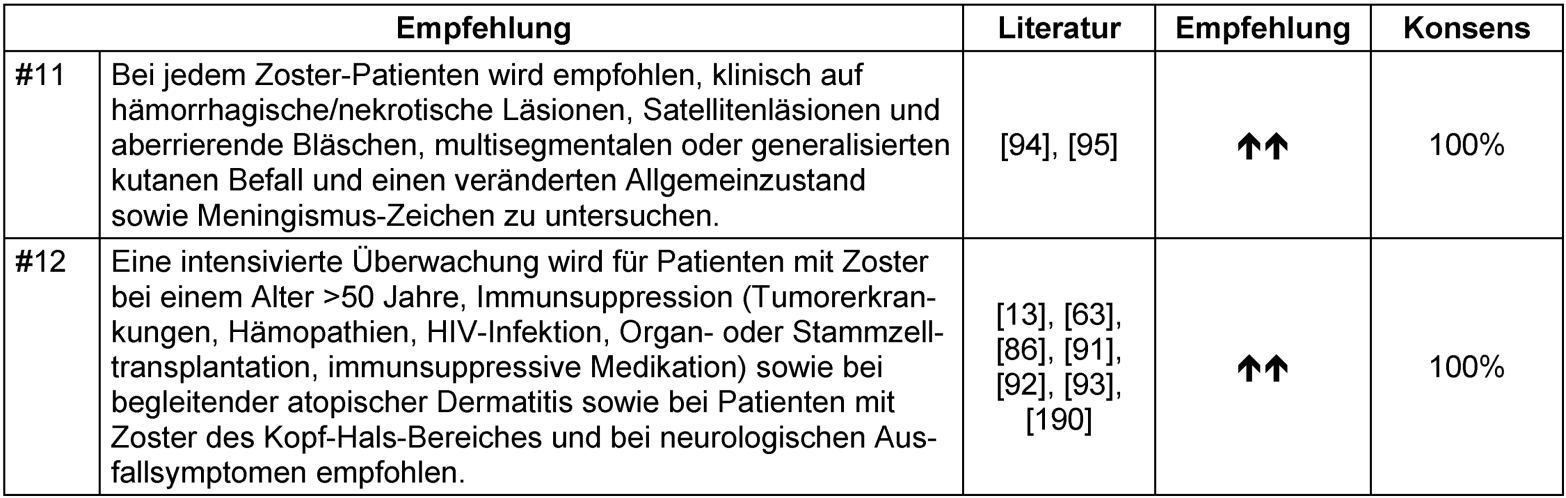
Tabelle 6: Diagnostische Empfehlungen bei Zoster; Empfehlung #11 und #12

**Tabelle 7:**
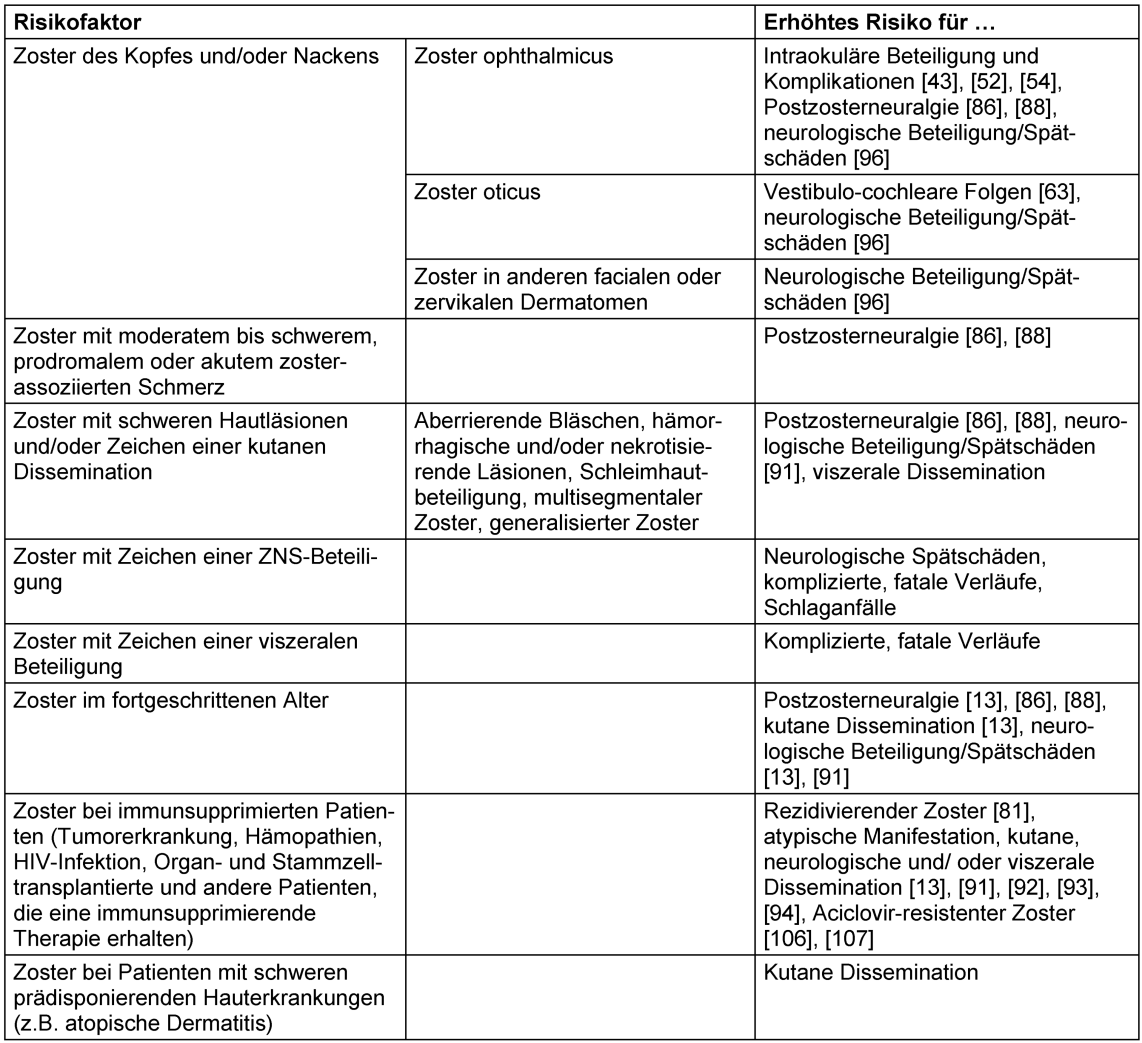
Tabelle 7: Risikofaktoren für komplizierte Verläufe des Zoster

**Tabelle 8:**
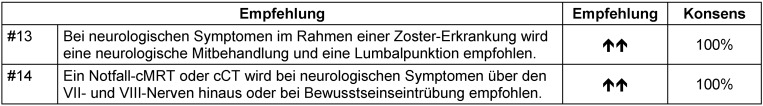
Tabelle 8: Diagnostische Empfehlungen bei Zoster; Empfehlung #13 und #14

**Tabelle 9:**
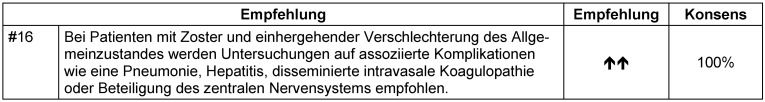
Tabelle 9: Diagnostische Empfehlungen bei Zoster; Empfehlung #16

**Tabelle 10:**
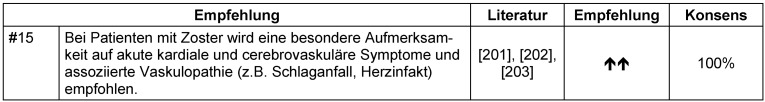
Tabelle 10: Diagnostische Empfehlungen bei Zoster; Empfehlung #15

**Tabelle 11:**
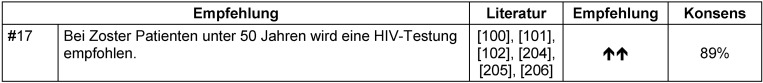
Tabelle 11: Diagnostische Empfehlungen bei Zoster; Empfehlung #17

**Tabelle 12:**
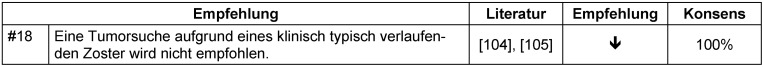
Tabelle 12: Diagnostische Empfehlungen bei Zoster; Empfehlung #18

**Tabelle 13:**
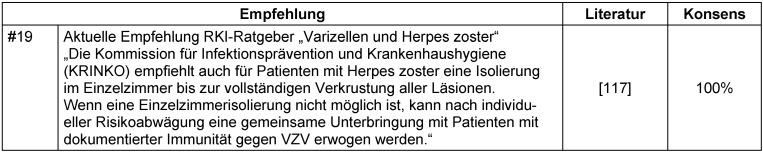
Tabelle 13: Empfehlung zur Isolation bei Zoster-Patienten; Empfehlung #19

**Tabelle 14:**
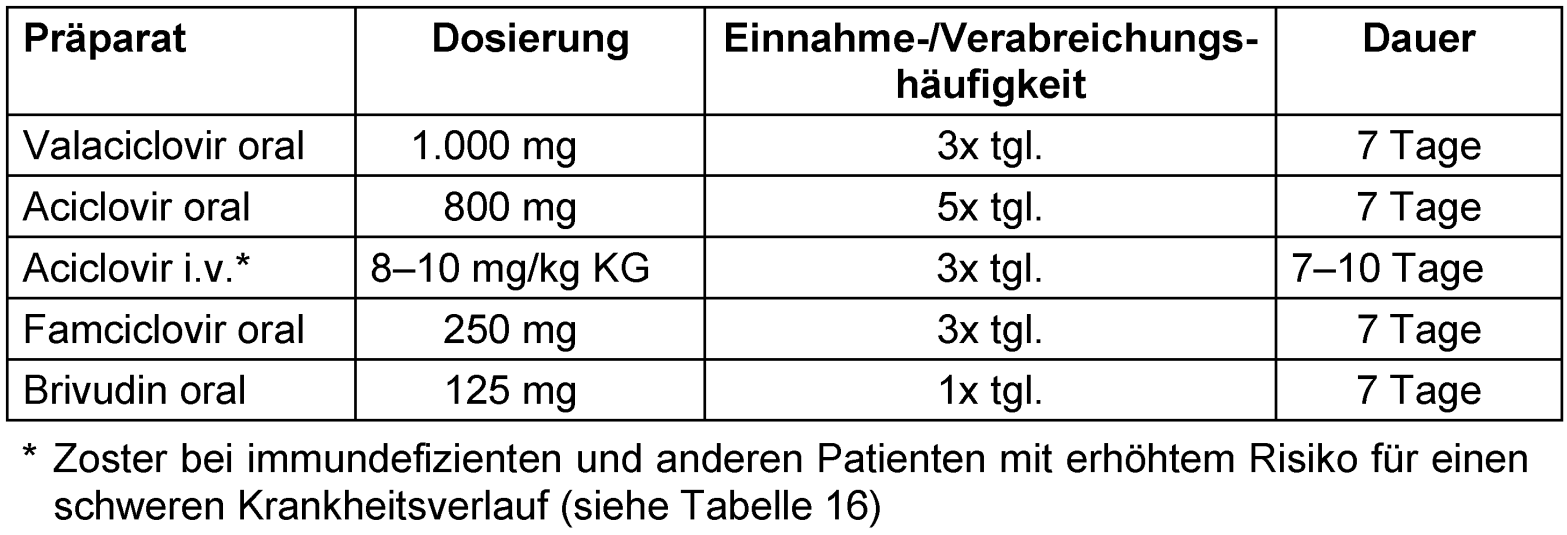
Tabelle 14: Übersicht über Dauer und Dosierung der antiviralen Standardsystemtherapie bei Zoster

**Tabelle 15:**
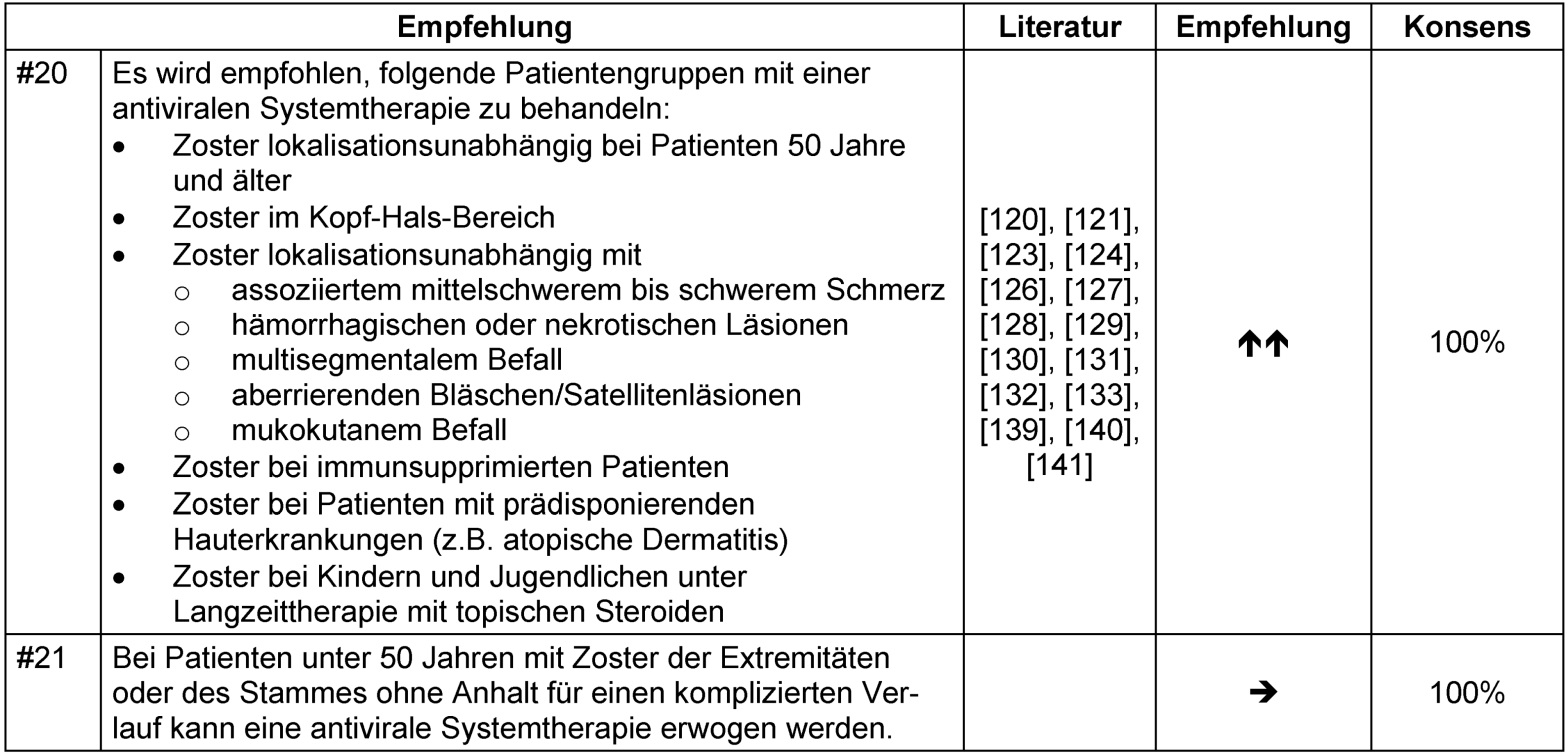
Tabelle 15: Therapie-Empfehlungen bei Zoster; Empfehlung #20 und #21

**Tabelle 16:**
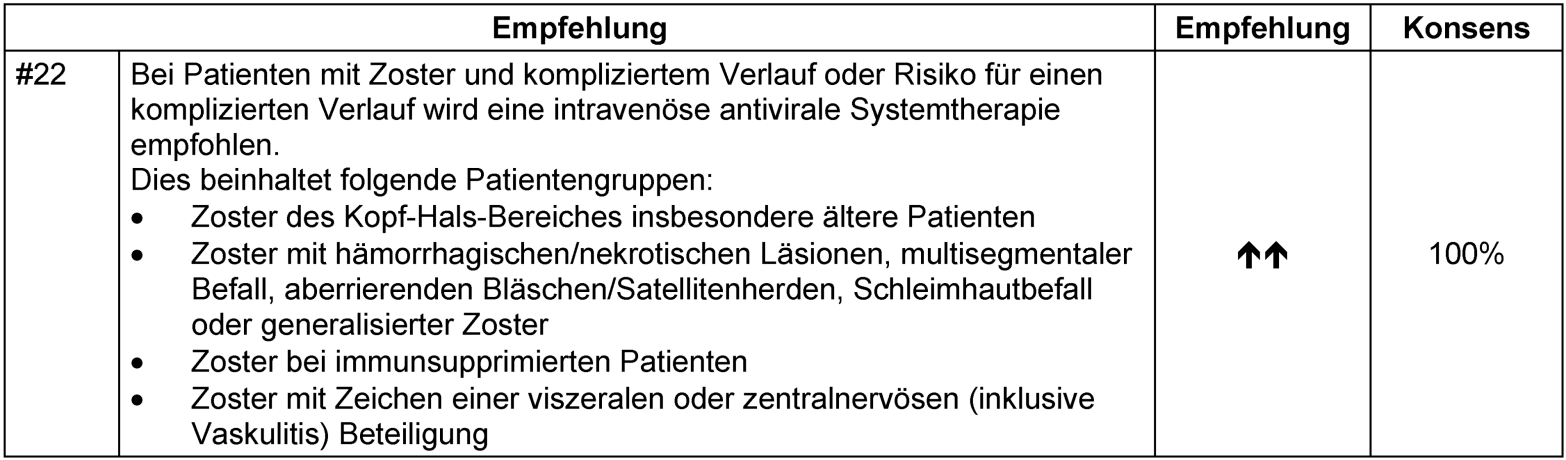
Tabelle 16: Therapie-Empfehlungen bei Zoster; Empfehlung #22

**Tabelle 17:**
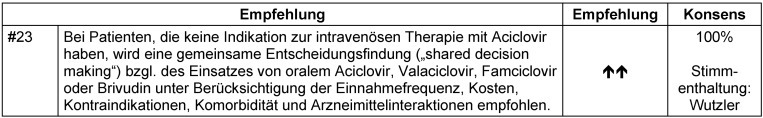
Tabelle 17: Therapie-Empfehlungen bei Zoster; Empfehlung #23

**Tabelle 18:**
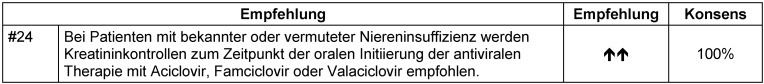
Tabelle 18: Therapie-Empfehlungen bei Zoster; Empfehlung #24

**Tabelle 19:**
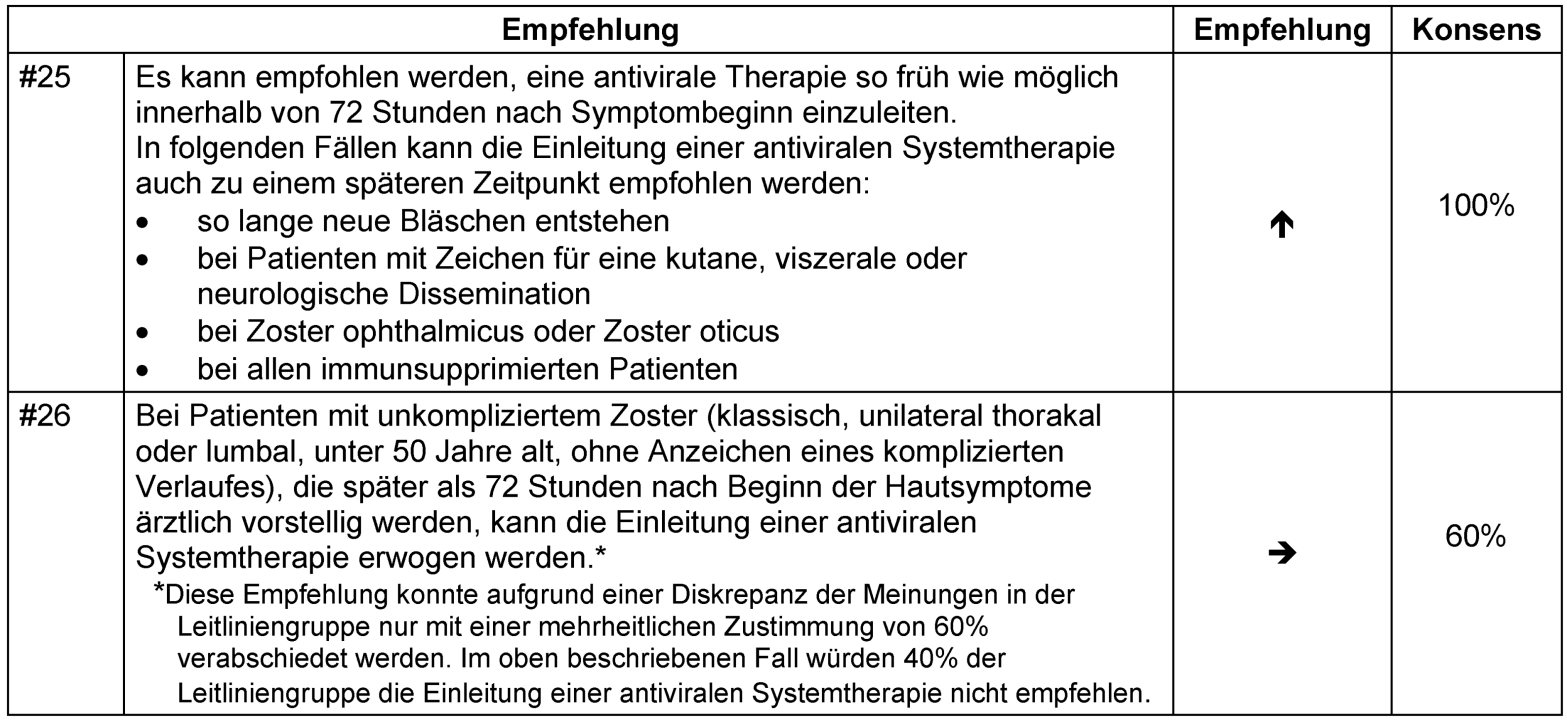
Tabelle 19: Therapie-Empfehlungen bei Zoster; Empfehlung #25 und #26

**Tabelle 20:**
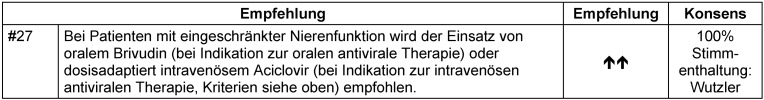
Tabelle 20: Therapie-Empfehlungen bei Zoster; Empfehlung #27

**Tabelle 21:**
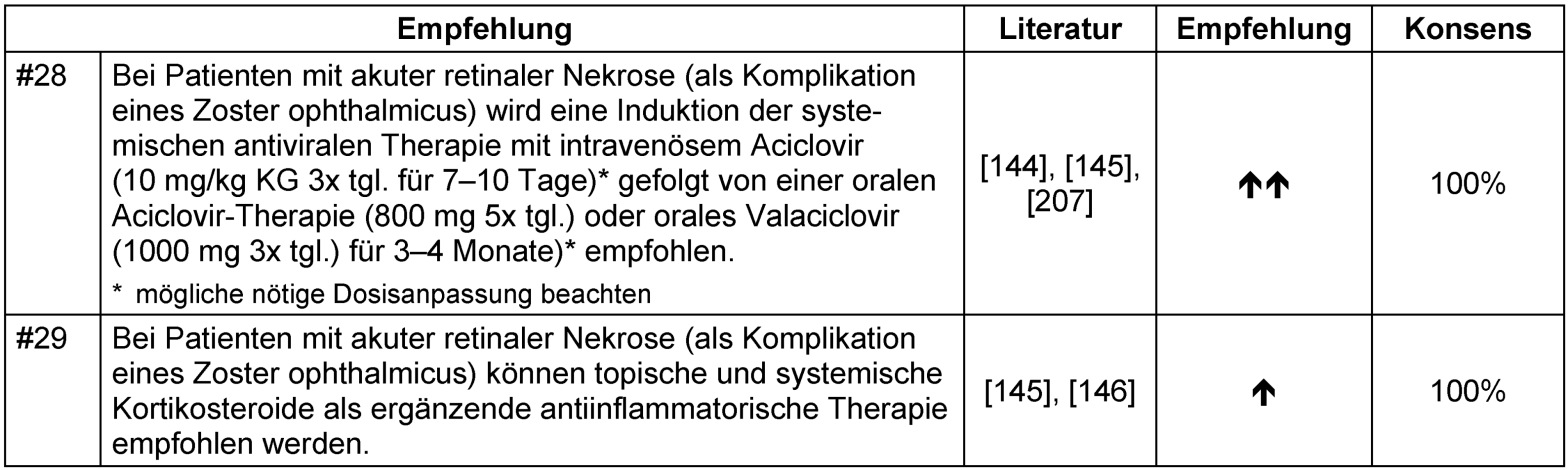
Tabelle 21: Therapie-Empfehlungen bei Zoster; Empfehlung #28 und #29

**Tabelle 22:**
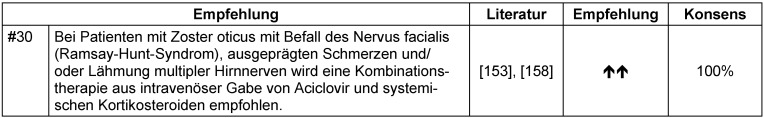
Tabelle 22: Therapie-Empfehlungen bei Zoster; Empfehlung #30

**Tabelle 23:**
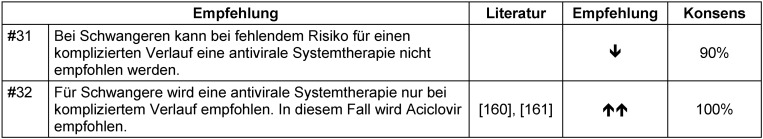
Tabelle 23: Therapie-Empfehlung bei Zoster; Empfehlung #31 und #32

**Tabelle 24:**
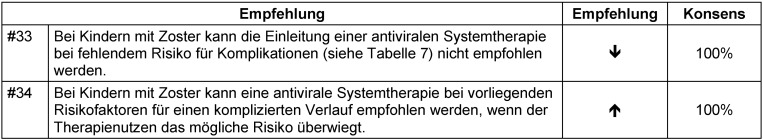
Tabelle 24: Therapie-Empfehlungen bei Zoster; Empfehlung #33 und #34

**Tabelle 25:**
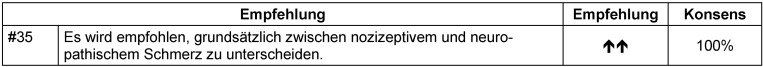
Tabelle 25: Empfehlung zur Schmerzunterscheidung bei Zoster; Empfehlung #35

**Tabelle 26:**
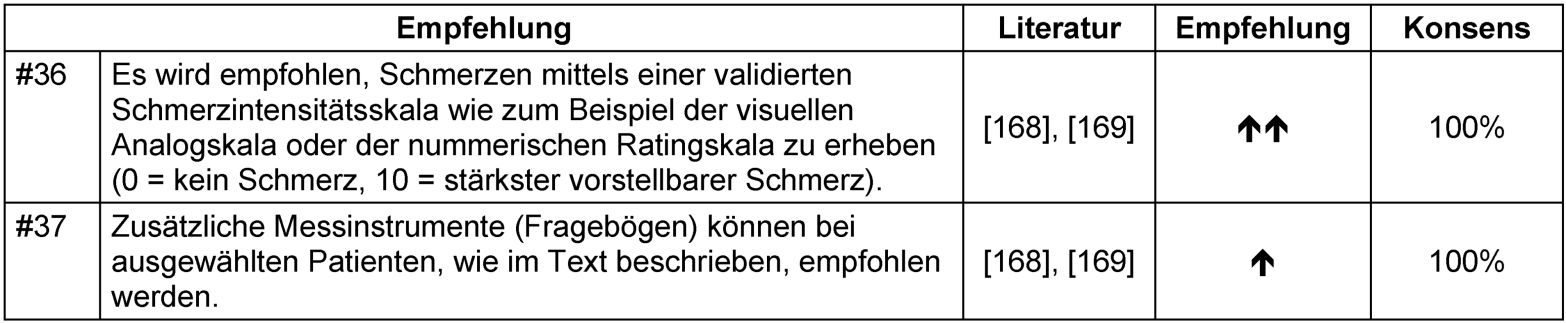
Tabelle 26: Empfehlungen zur Schmerzbehandlung bei Zoster; Empfehlung #36 und #37

**Tabelle 27:**
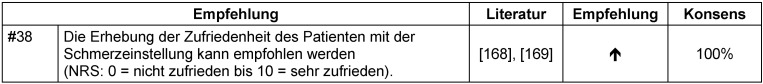
Tabelle 27: Empfehlungen zur Schmerzbehandlung bei Zoster; Empfehlung #38

**Tabelle 28:**
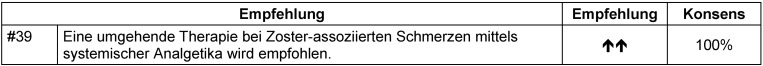
Tabelle 28: Empfehlungen zur Schmerzbehandlung bei Zoster; Empfehlung #39

**Tabelle 29:**
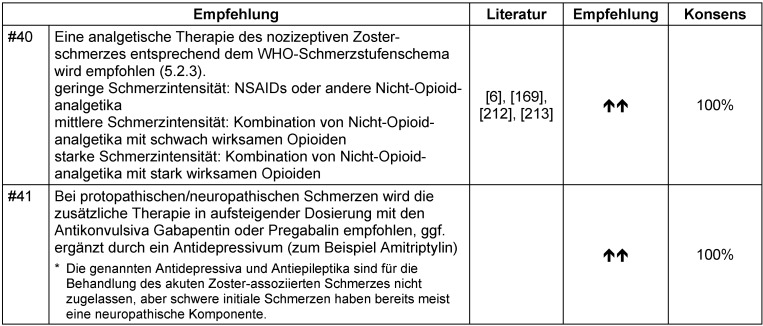
Tabelle 29: Empfehlungen zur Schmerzbehandlung bei Zoster; Empfehlung #40 und #41

**Tabelle 30:**
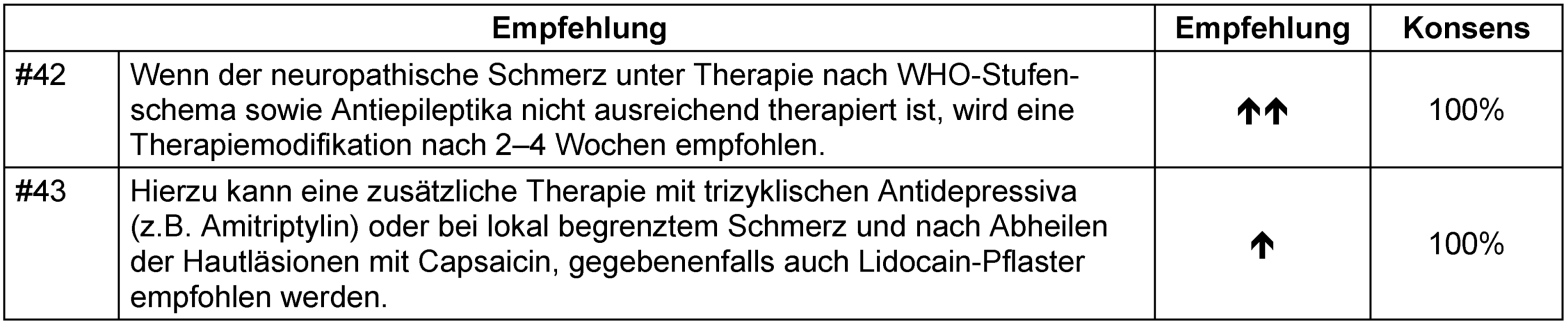
Tabelle 30: Empfehlungen zur Schmerzbehandlung bei Zoster; Empfehlung #42 und #43

**Tabelle 31:**
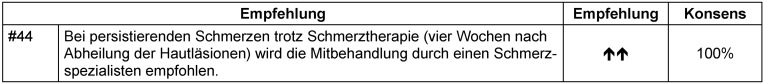
Tabelle 31: Empfehlungen zur Schmerzbehandlung bei Zoster; Empfehlung #44

**Tabelle 32:**
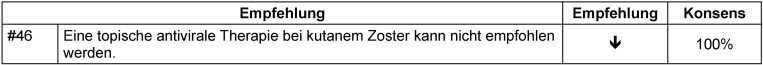
Tabelle 32: Empfehlungen zur topischen Therapie bei Zoster; Empfehlung #46

**Tabelle 33:**
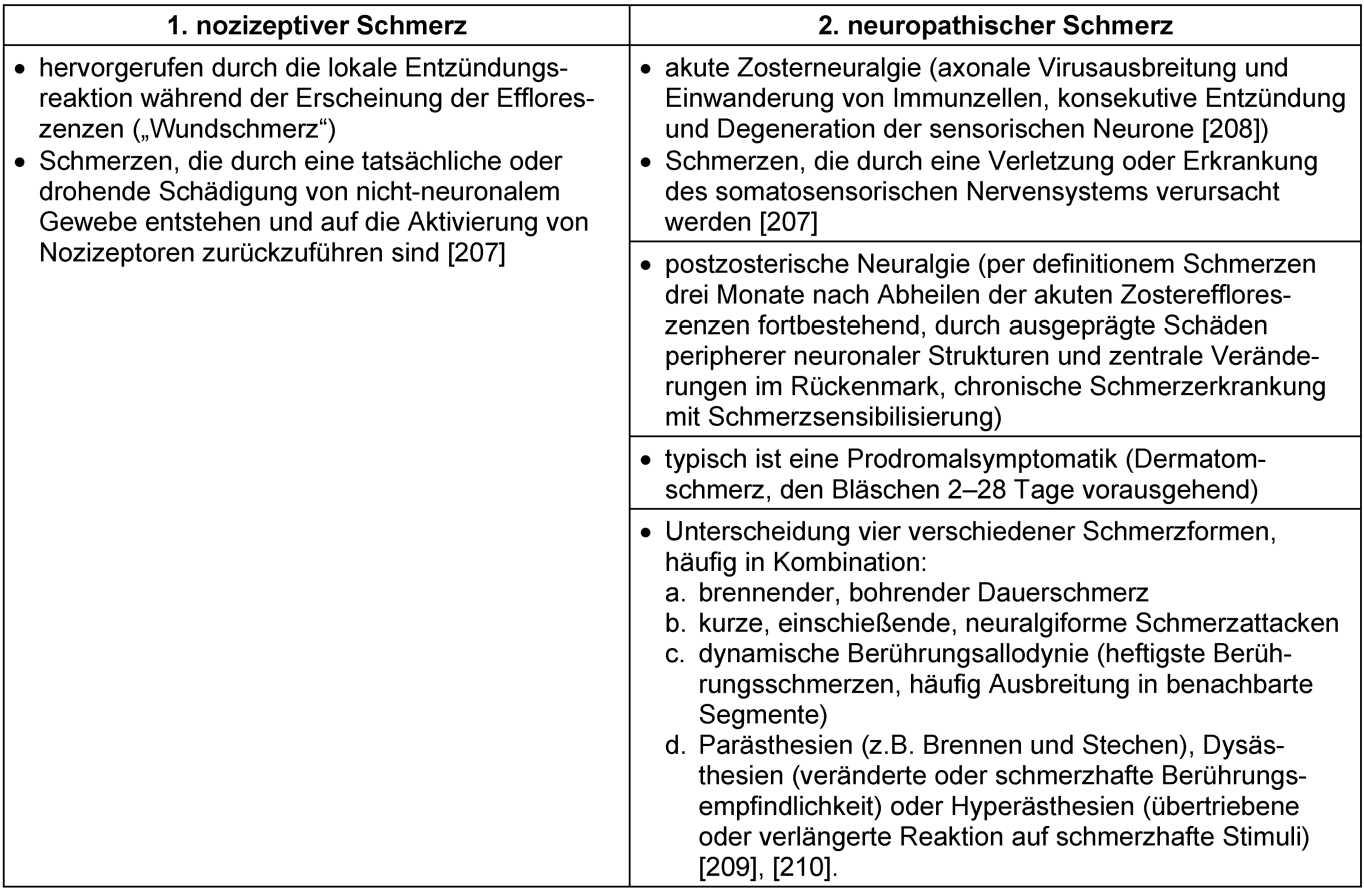
Tabelle 33: Definition des nozizeptiven und neuropathischen Schmerzes

**Tabelle 34:**
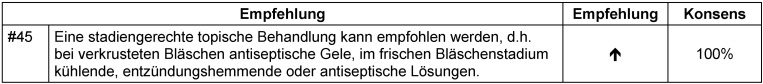
Tabelle 34: Empfehlungen zur topischen Therapie bei Zoster; Empfehlung #45

**Tabelle 35:**
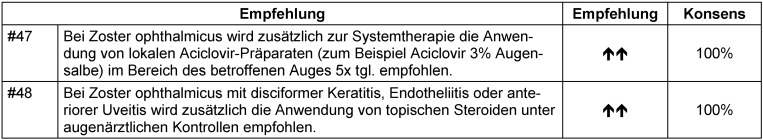
Tabelle 35: Empfehlungen zur topischen Therapie bei Zoster; Empfehlung #47 und #48

**Tabelle 36:**
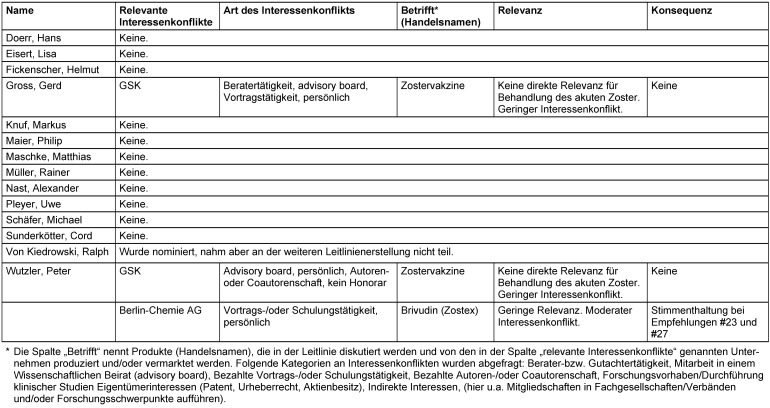
Tabelle 36: Darstellung relevanter Interessenkonflikte und des Umgangs mit selbigen
